# Genome-Wide DNA Methylation Analysis Reveals Phytoestrogen Modification of Promoter Methylation Patterns during Embryonic Stem Cell Differentiation

**DOI:** 10.1371/journal.pone.0019278

**Published:** 2011-04-29

**Authors:** Noriko Sato, Naomi Yamakawa, Moe Masuda, Katsuko Sudo, Izuho Hatada, Masaaki Muramatsu

**Affiliations:** 1 Department of Molecular Epidemiology, Medical Research Institute, Tokyo Medical and Dental University, Tokyo, Japan; 2 Research Team for Geriatric Disease, Institute of Gerontology and Tokyo Metropolitan Geriatric Hospital, Tokyo, Japan; 3 Animal Research Center, Tokyo Medical University, Tokyo, Japan; 4 Laboratory of Genome Science, Biosignal Genome Resource Center, Institute for Molecular and Cellular Regulation, Gunma University, Maebashi, Gunma, Japan; Baylor College of Medicine, United States of America

## Abstract

**Background:**

Environmental challenges during development affect the fetal epigenome, but the period(s) vulnerable to epigenetic dysregulation is(are) not clear. By employing a soy phytoestrogen, genistein, that is known to alter the epigenetic states of the *A^vy^* allele during embryogenesis, we have explored the sensitive period for epigenetic regulation. The post-implantation period, when *de novo* DNA methylation actively proceeds, is amenable to *in vitro* analysis using a mouse embryonic stem (ES) cell differentiation system.

**Methods and Findings:**

Mouse ES cells were differentiated in the presence or absence of genistein, and DNA methylation patterns on day 10 were compared by microarray-based promoter methylation analysis coupled with a methylation-sensitive endonuclease (HpaII/McrBC)-dependent enrichment procedure. Moderate changes in methylation levels were observed in a subset of promoters following genistein treatment. Detailed investigation of the *Ucp1* and *Sytl1* promoters further revealed that genistein does not affect *de novo* methylation occurring between day 0 and day 4, but interferes with subsequent regulatory processes and leads to a decrease in methylation level for both promoters.

**Conclusion:**

Genistein perturbed the methylation pattern of differentiated ES cells after *de novo* methylation. Our observations suggest that, for a subset of genes, regulation after *de novo* DNA methylation in the early embryo may be sensitive to genistein.

## Introduction

In mammals, the intrauterine environment affects the fetal epigenome. For example, maternal dietary genistein increases the level of DNA methylation at the cryptic promoter derived from intracisternal A-particle insertion in the *agouti* gene (the viable yellow allele of *agouti* is denoted *A^vy^*) [1]. This genistein-induced hypermethylation of *A^vy^* occurs to a similar extent in tissues from all three germ layers (brain, liver, and kidney) and persists into adulthood. Because *A^vy^* undergoes epigenetic reprogramming after fertilization [2], alteration of DNA methylation patterns by genistein would probably occur within the developmental window after fertilization but before germ layer differentiation [3].

During early embryonic development, approximately at implantation, the DNA methylation patterns of individual promoters dynamically change. For example, the methylation of specific sites in *Apoa1* is erased after fertilization; after implantation, methylation is restored by the gastrulation stage [4]. Recent genome-wide analysis of promoter DNA methylation in the early embryo has confirmed that *de novo* methylation occurs during implantation in 476/691 promoters that are methylated in the E9.5 embryo [5]. Of importance, DNA methylation patterns established around implantation can be modified by further methylation or demethylation during subsequent stages of organogenesis [5]. However, the influence of environment on DNA methylation dynamics of intrinsic promoters is still unknown.

To clarify how environmental factors affect DNA methylation dynamics, especially during the post-implantation period, we characterized the alterations in DNA methylation patterns induced by genistein in an embryonic stem (ES) cell differentiation system. We developed a novel DNA methylation assay, MspI fragment-based DNA methylation typing (MFMT), for use in combination with a mouse promoter microarray to identify genistein-mediated differentially methylated regions (DMRs). Of the 149 DMRs identified, 54 regions were more methylated and 95 regions were less methylated in genistein-treated cells than in control cells. Among these DMRs, 74 regions were located proximal to the transcriptional start site (TSS) of adjacent gene(s). The genistein-mediated hypomethylation of the *Ucp1* and *Sytl1* promoters was further analyzed for the effects of genistein on methylation dynamics. Bisulfite sequencing confirmed the decrease in methylation levels of both promoters on day 10. Of interest, the methylation levels of the *Ucp1* and *Sytl1* promoters peaked on day 4, and peak levels were equivalent in genistein-treated and untreated cells. Thus, once *de novo* DNA methylation of these promoters was complete, genistein appeared to accelerate the subsequent decline in methylation levels. Our results suggest that genistein influences DNA methylation established around the post-implantation period, which may partly explain the vulnerability of the embryonic epigenome to intrauterine environmental changes.

## Materials and Methods

### ES cell culture and differentiation

ES cells were maintained in Dulbecco's Modified Eagle Medium (Sigma, St. Louis, MO) with 15% fetal calf serum (Millipore, Billerica, MA), 10^3^ U ml^−1^ leukemia inhibitory factor, 0.1 mM MEM non-essential amino acids (Sigma), 1× nucleosides mix, 2 mM L-glutamine, penicillin/streptomycin, and 40 µM 2-mercaptoethanol (Sigma). ES cell lines CCE (strain 129/Sv) and E14Tg2a (strain 129/Ola), gifts of Dr. A. Miyajima (University of Tokyo), are feeder-independent. For ES cell differentiation, embryoid body (EB) formation was induced by hanging-drop culture without leukemia inhibitory factor for 2 days, followed by 2 days of suspension culture and 6 days of culture on gelatinized dishes. During differentiation, ES cells were treated with the indicated concentrations of genistein (0, 1, 5, or 10 µM) dissolved in dimethyl sulfoxide (DMSO); the medium was replaced every other day. The final DMSO concentration in all the samples was 0.01% (v/v). From differentiation day 8 to day 12, the proportion of EBs with contracting myocardiocytes was evaluated. Concentrations of up to 5 µM genistein did not impair cardiac differentiation (Figure S1).

### Real-time PCR analysis

*Esr1*, *Esr2*, *Pou5f1*, *T*, *Fgf5*, *Wnt3a*, *Pdgfra*, *Tbx5*, *Gata4*, and *Gata6* mRNA levels were measured by real-time RT-PCR in a LightCycler 480 (Roche Applied Science, Mannheim, Germany). Total RNA was extracted from ES cells (day 0, day 4 of control, day 4 of genistein-treated, day 10 of control, and day 10 of genistein-treated) using the RNeasy mini kit (QIAGEN, Hilden, Germany). After treatment with DNase I (Invitrogen, Carlsbad, CA), cDNA was synthesized from 1 µg RNA per reaction with 200 U Superscript III, 2.5 µM oligo (dT)_20_, 0.5 mM dNTP, 5 mM MgCl_2_, and 10 mM DTT in reaction buffer (Invitrogen) at 50°C for 50 min. All reactions were run together with RT minus controls. After termination of cDNA synthesis at 85°C for 5 min, the reaction mixture was further incubated with RNase H at 37°C for 20 min. Real-time PCR was performed using the LightCycler 480 Probe Master kit (Roche Applied Science) and the specific primers and the corresponding Universal Probe Library probe (Roche Applied Science) according to the manufacturer's instructions. The identification numbers of Universal Probe Library probes and primer sequences are listed in Table S1. Normalized gene expression values, calibrated with the value on day 0, were obtained with LightCycler Relative Quantification software (ver 1.5, Roche Applied Science) using *Actb* as a reference gene (Figures S2 and S8).

### MspI fragment-based DNA methylation typing (MFMT): assay and analysis

Genomic DNA was extracted from control cells (DMSO only) and genistein-treated cells at day 10 of CCE differentiation using the QIAamp DNA Mini kit (QIAGEN). A total of 200 ng of each DNA sample was digested to completion for 3 h with 10 U MspI, and the digested DNA was purified with the MinElute Reaction Clean-up kit (QIAGEN). Two complementary oligonucleotides (5′-AGCACTCTCCAGCCTCTCACCGAC-3′ and 5′-CGGTCGGTGAGAGGCTGG-3′) were annealed and ligated to the MspI fragments, and their 3′ ends were extended with Platinum Taq DNA polymerase (Invitrogen, Carlsbad, CA) at 72°C for 15 min. The reaction mixture was then divided into three aliquots and subjected to enzymatic digestion for 4 h. One aliquot was treated with 10 U MspI, another with 10 U HpaII, and the third aliquot with 10 U McrBC (Figure S3). Ligation-mediated PCR (LM-PCR) was carried out for each product with the FastStart High Fidelity PCR System (Roche, Mannheim, Germany) in buffer containing 1.8 mM MgCl_2_, 2% DMSO, and the specific primer 5′ -GCCTCTCACCGACCGG-3′. Amplification conditions consisted of a hot start at 95°C for 2 min, followed by 26 cycles of 95°C for 20 s, 72.9°C for 20 s, and 73°C for 30 s; 18 cycles of 95°C for 20 s, 72.3°C for 20 s, and 73°C for 10 s; 8 cycles of 95°C for 20 s, 71.0°C for 20 s, and 73°C for 30 s; and finally a hold at 72°C for 5 min. The quality of the LM-PCR reaction was tested with the Agilent 2100 bioanalyzer (Santa Clara, CA). PCR products were purified with a MinElute Reaction Clean-up kit, and were labeled with Alexa 555 or 647 using the BioPrime Plus Array CGH Genomic Labeling System (Invitrogen) in accordance with the manufacturer's instructions, with the exception of a use of 100 U Klenow fragment per labeling reaction. The labeled PCR products were mixed and then hybridized to the NimbleGen (Madison, WI) mouse 385K CpG Island Plus Promoter Array (cat# 00893), according to the manufacturer's protocol. Hybridized arrays were scanned with a NimbleGen MS200 Scanner, and the images were analyzed with NimbleScan software (ver 2.6).

The NimbleGen mouse 385K promoter array contains 373,638 probes in total on average ∼1.3 kb of upstream sequence and ∼0.5 kb of downstream sequence from the TSS, covering 15,963 promoters/CpG islands. Each probe is 50–70 nucleotides in length, with roughly 18 probes per region with 100–bp spacing. We selected a subset of probes for our analysis (Figure S3). First, we excluded the probes that contain the MspI recognition sequence (CCGG). Next, because the LM-PCR products were generated mainly in the 100–1000 bp size range, we focused on probes enclosed by two MspI sites with an interval length between 100 and 1000 bp (Figure S3). Our selection criteria led to the retention of 127,857 (34.2% of the total NimbleGen probes) for further analysis.

If the CpG at the end of the MspI-digested fragment is methylated, the adaptor-ligated product remains resistant to HpaII digestion, so that the LM-PCR product (the HpaII-resistant product) can be fully generated (Figure S3). If the MspI fragment contains McrBC target CpGs (recognition sequence R^m^C(N)_55–103_ R^m^C) and those CpGs are methylated, the ligation product is sensitive to McrBC digestion, leading to infrequent generation of the LM-PCR product (McrBC-resistant product). Using the ChIP Compute Ratio Files Analysis in the NimbleScan software, the log_2_ (HpaII^resistant(r)^/McrBC^resistant(r)^) values were calculated for individual probes. During this process, the values were scaled to zero by subtracting the bi-weight mean for all features on the array from each log_2_ ratio. The log_2_ (HpaII^r^/McrBC^r^) value for each probe reflects the methylation level of the MspI fragment in which the probe exists.

To evaluate the methylation detection performance of the MFMT assay, we calculated the mean log_2_ (HpaII^r^/McrBC^r^) values for the MspI fragments in several methylation-control promoters. Because there was no precise information about the promoter DNA methylation levels in day-10 differentiated ES cells, we searched the possible candidates for universally (in somatic cells) methylated or unmethylated promoter regions by using previously published data [6]. For selection of the methylated regions, we considered three criteria as follows: (1) mean methylation levels assayed by RRBS (reduced representation bisulfite sequencing) were high (>0.30) in ES, NPCs (neural precursor cells), astrocytes, whole brain, CD4+ T cells, CD8+ T cells, B cells, MEFs (mouse embryonic fibroblasts), tail-tip, liver, spleen, and lung ([6]); (2) a germ-line–specific promoter was present; and (3) the promoter region contained a pair of adjoining MspI sites with an interval that was long enough to contain more than two consecutive probes of the microarray and short enough for LM-PCR to achieve the MFMT assay. Eventually, seven regions of *Dazl*, *Sycp1*, *Tuba3a*, *Spo11*, *Hormad1*, *AU022751*, and *Sycp2* were selected as “methylation positive controls.” On the other hand, for the unmethylated regions, we considered three criteria as follows: (1) mean methylation levels assayed by RRBS were low (<0.20) in all the samples analyzed by Meissner *et al.* [6]; (2) there was a ubiquitously expressed gene promoter; and (3) the promoter or CpG island region contained a pair of adjoining MspI sites with an interval length that was adequate for the MFMT assay. Although over 2000 gene promoters met two criteria, such as (1) and (2), less than half met a third criterion because of so-called HpaII Tiny Fragments (HTFs) ([7]). Among these, we selected 46 regions that are highly transcribed in ES cells as “methylation negative controls.” Figure S4 shows the scatter plot of the mean log_2_ (HpaII^r^/McrBC^r^) values of untreated and genistein-treated samples for these methylation control regions. The blue dots represent methylation-positive whereas the red dots indicate methylation-negative controls. Figure S5 shows the detailed information about the four representatives of these control regions (*Dazl*, *Spo11*, *Dhfr*, and *Psmd1*). Thus, the mean log_2_ (HpaII^r^/McrBC^r^) values derived from the MFMT analysis were substantially related to the CpG methylation levels, with larger values reflecting higher methylation levels. Additionally, little inter-microarray variability was observed among the log_2_ (HpaII^r^/McrBC^r^) values in the methylation-control regions, consistent with a Pearson correlation coefficient of 0.923 for all log_2_ (HpaII^r^/McrBC^r^) probe values from the control and 5 µM genistein-treated samples. All the statistical computation and graphics were performed using the R Statistical Package (http://www.r-project.org/). Gene ontology analysis was conducted using the Database for Annotation, Visualization and Integrated Discovery (DAVID) v6.7 [8].

### Bisulfite sequencing

One microgram of genomic DNA from ES cells and tissues was bisulfite converted with an EpiTect Bisulfite Kit (QIAGEN) using a standard protocol. Bisulfite conversion of embryonic genomic DNA was performed with the EZ DNA Methylation-Direct Kit (Zymo Research, Orange, CA). Bisulfite sequencing primers are shown in Table S2. PCR products were cloned into the pT7Blue T-vector (Novagen, Madison,WI) and transformed into *Escherichia coli*. Plasmid DNA from positive colonies was purified and sequenced at the Tokyo Medical and Dental University Genome Analysis Facility (Tokyo, Japan). Sequence and statistical analyses were performed with the QUantification tool for Methylation Analysis; http://quma.cdb.riken.jp/top/quma_main_j.html [9]. The statistical significance of the difference between two bisulfite sequence groups at each CpG site was evaluated with the Fisher's exact test.

### Mouse embryos and adult tissues

All animal work was conducted in accordance with our institution's ethical guidelines for animal experiments. The protocols for animal handling and treatment were reviewed and approved (approval ID: S-22038) by the Animal Care and Use Committee at Tokyo Medical University (Tokyo, Japan). Oocytes were collected from the oviducts of C57BL/6J unmated female mice that were treated for superovulation by intraperitoneal injection of 5 U of pregnant mare serum gonadotropin followed 48 hr later by 5 U human chorionic gonadotropin. Twenty hours after the second injection, oocytes were collected and fertilized *in vitro* with C57BL/6J sperm. Zygotes were cultured in drops containing M16 medium covered with mineral oil. To obtain E6.75 and E10 embryos, two-cell embryos were transferred into day-0.5 pseudo-pregnant ICR female mice the next day. For the E6.75 stage, seven embryos were collected and genomic DNA was isolated. For the E10 stage, genomic DNA samples were isolated separately from two independent embryos. Genomic DNA was isolated from adult mouse liver and lung with the QIAamp DNA Mini Kit (QIAGEN).

## Results

### Identification of genistein-mediated DMRs

By day 10 of differentiation, ES cells give rise to a variety of cells, including contracting cardiomyocytes. Exposure to 5 µM genistein did not affect cardiomyocyte differentiation of ES cells, indicating that treatment is not toxic (Figure S1). The analysis of differentiation marker expression also indicated that 5 µM genistein did not impair overall ES cell differentiation (Figure S2). The global DNA methylation patterns in differentiated ES cells with or without genistein treatment were analyzed with the MFMT assay, which involves using either HpaII or McrBC to dissect the methylation status of the end or interior CpGs for every MspI fragment, respectively (Figure S3). Methylation levels are reflected in the log_2_ (HpaII^resistant(r)^/McrBC^resistant(r)^) value for each microarray probe (see Materials and Methods for details).

**Figure 1:**
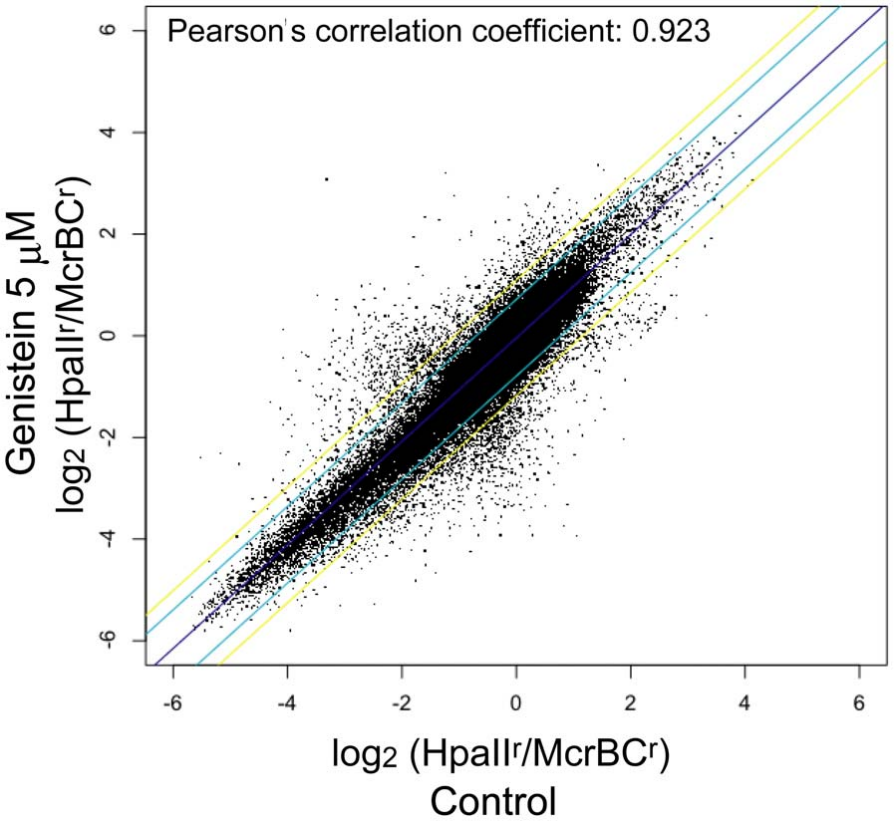
Scatter plot of the log_2_ (HpaII^r^/McrBC^r^) values of control and genistein-treated samples for individual probes. The total number of probes used in the MspI fragment-based DNA methylation typing assay is 127,857. The log_2_ (HpaII^r^/McrBC^r^) values for the individual probes of dimethyl sulfoxide-treated (control) and genistein-treated samples were plotted. There was little variability in the log_2_ (HpaII^r^/McrBC^r^) values of the same probe between the two samples. The regression line is purple; the cyan and yellow lines denote 2 and 3 standard deviations, respectively, of the horizontal distance from the regression line.

Genistein-mediated global changes in methylation levels were assessed by the difference of the log_2_ (HpaII^r^/McrBC^r^) values between genistein-treated and untreated (control) cells; a high linear correlation was detected (Pearson correlation coefficient, 0.923) for the individual probes between the two datasets (Figure 1). The log_2_ (HpaII^r^/McrBC^r^) values in the methylation control regions were also similar in the control and genistein-treated cells (Figures S4 and S5). Thus, although genistein treatment did not alter the DNA methylation levels in most regions detected by this assay, a subset of loci showed significant differences in their methylation levels.

Next, the difference in the log_2_ (HpaII^r^/McrBC^r^) values for each probe was calculated between the control and genistein-treatment datasets (mean = 0.048, standard deviation = 0.379; Figure S6). We selected probes with log_2_ (HpaII^r^/McrBC^r^) differences larger than the mean consecutive difference; of these probes, 1330 probes indicated regions hypomethylated following genistein treatment (values above the upper limits of the mean consecutive difference; mean plus three standard deviations), whereas 952 probes reflected hypermethylation of regions following genistein treatment (values below the lower limits of the mean consecutive distance; mean minus three standard deviations). However, the most pertinent methylation differences are represented by the same directional log_2_ (HpaII^r^/McrBC^r^) changes across multiple probes that are in close proximity to one another on the genome; thus, we identified loci with more than two differentially methylated probes. Out of 1330 hypomethylation and 952 hypermethylation probes, 223 (16.7%) and 117 (12.3%) probes, respectively, comprised sets of consecutive probes within the same MspI fragment or adjoining MspI fragments. Therefore, we regarded those MspI fragments as genistein-mediated DMRs (Figure S7).

We identified a total of 149 DMRs, of which 54 regions were more methylated and 95 regions were less methylated in genistein-treated cells than in control cells (Table 1). Approximately half of these DMRs (48.3%) were located in promoters, while the other half (50.3%) occurred in gene-body regions. Fifty-one DMRs were located in proximal promoter regions (less than 200 bp from TSS), nine of which contained bi-directional promoters. We performed gene ontology analysis using the DAVID Functional Annotation tool for all 155 genes contained in DMRs, excluding one non-coding RNA and two non-genic regions. Thirty-eight genes (24.5%) were related to “gene expression” in the Gene Ontology category (p<2.3×10^−4^), while 25 (16.1%) and 7 genes (4.5%) were related to “embryo” (p<0.016) and “fetal brain” (p<0.036), respectively, in the Tissue_Expression category (Table S3). The indicated p-values correspond to modified Fisher's exact p-values for gene-enrichment analysis (EASE score). No further specific category was enriched in the genistein-mediated DMRs.

**Table 1:** Details of the relative positions of detected GEN-mediated DMRs.

	Regions with decreased methylation levels after GEN exposure	Regions with increased methylation levels after GEN exposure
More than 200 bp upstream of nearest TSS	15	6
Less than 200 bp upstream of nearest TSS	32	19
In gene-body regions	47 (16)*	28 (7)*
In intergenic regions	1	1
**Total Number**	**95**	**54**

**GEN**, genistein; **DMR**, differentially methylated region; **TSS**, transcription start site;

*number of DMRs located in exon 1.

Seventy-four DMRs (mean length ∼290 bp) were located in proximal promoters and the first exon of genes (Tables 2–5). In general, the sequences of the regions close to TSSs are relatively conserved between human and mouse [10]. Furthermore, bisulfite sequencing across matched tissues from human and mouse revealed strongly conserved patterns of DNA methylation in CpG island regions of *SHANK3* promoter regions (including alternative promoters) [11]. Therefore, for 64 DMRs CpG methylation data from the corresponding human regions were acquired from the available public resources [11], [12]. Most DMRs contained High CpG-density promoters (HCPs), and the somatic methylation levels of their human counterparts were “unmethylated.” Additionally, 10 intermediate CpG-density promoters (ICPs) and 7 low CpG-density promoters (LCPs) were found in genistein-mediated DMRs, with the somatic methylation levels of the human counterparts varying from “unmethylated” to “methylated”, including four counterparts (*Ucp1*, *Sytl1*, *Hspb3*, and *Ripk3*) with “intermediate” methylation levels. Because ICPs and LCPs are thought to be primary targets for DNA methylation [5], these four promoters could possibly be DNA methylated as well in mouse cells. This possibility prompted us to address the question of how genistein influences DNA methylation levels of ICP and LCP promoters in the early embryonic stages.

**Table 2:** Methylation levels of 32 DMRs in promoters that decreased following GEN exposure.

Position	Gene(s)	Control	GEN	DM/T	Class	Ref.
chr4:134392301–134392772	Runx3	2.01±0.58	0.28±0.07	4/4	LCP	M
chr18:24132054–24132434	Zfp35	1.78±0.33	−0.01±0.35	3/3	ICP	ND
chr12:84958869–84959299	Acot5	1.17±0.40	−0.01±0.39	3/4	HCP	ND
chr18:78758937–78759113	Slc14a2	0.70±0.76	−1.19±0.02	2/2	LCP	ND
chr14:53641194–53641417	Acin1-1700123O20Rik	0.38±0.18	−3.36±0.03	2/2	HCP	U
chr10:80787136–80787377	Dohh	0.35±0.54	−1.29±0.37	2/2	HCP	ND
chr15:41619452–41619822	Oxr1	0.19±0.13	−1.66±1.37	2/3	HCP	U
chr1:36218070–36218359	Neurl3	0.01±0.66	−1.45±0.74	2/3	LCP	ND
chr4:132534712–132535246	Sytl1	−0.03±0.48	−1.13±0.44	4/5	LCP	I
chr12:45080426–45080612	Dnajb9	−0.11±0.25	−2.82±0.70	2/2	HCP	U
chr1:12703475–12703685	Sulf1	−0.16±0.54	−3.19±0.53	2/2	LCP	U
chr17:31895678–31896022	Notch3	−0.18±0.13	−2.04±0.05	3/3	HCP	I
chr18:38727246–38727472	Spry4-9630014M24Rik	−0.20±0.54	−1.75±0.05	2/2	HCP	U
chr13:74674934–74675272	Exoc3	−0.26±0.56	−1.53±0.81	3/3	HCP	ND
chr15:84059440–84059681	Parvb	−0.33±0.42	−2.38±0.22	2/2	HCP	U
chr9:122799448–122799842	Kif15-1110059G10Rik	−0.34±0.17	−1.96±0.65	3/4	HCP	U
chr14:101869393–101869657*	Kctd12	−0.34±0.63	−2.04±0.71	2/2	HCP	U
chr4:133976469–133976747	Man1c1	−0.41±0.14	−1.86±0.62	2/2	HCP	U
chr14:55525969–55526381	Cenpj	−0.48±0.13	−1.85±0.51	3/3	HCP	U
chr19:6235862–6236239	Ppp2r5b	−0.50±0.31	−2.56±0.37	3/3	HCP	I
chr16:43809152–43809407	2610015P09Rik-Qtrtd1	−0.56±0.28	−2.36±0.11	2/2	ICP	U
chr4:61988560–61988857	Hdhd3	−1.03±0.11	−2.06±0.78	2/3	HCP	U
chr9:123566265–123566427	Lztfl1	−1.08±0.04	−2.54±0.08	2/2	HCP	U
chr5:139738113–139738428	Zfand2a	−1.15±0.93	−2.50±1.03	2/2	HCP	U
chr7:138778679–138778997	Jakmip3	−1.34±0.14	−2.75±0.31	3/3	HCP	U
chrX:142800291–142800547	Lrch2	−1.61±0.22	−3.23±0.40	2/2	HCP	U
chr6:87777440–87777770	Rab43	−1.69±0.97	−2.91±1.33	2/3	HCP	I
chr8:86180062–86,180,386	Ucp1	−1.78±0.66	−3.05±0.47	3/3	ICP	I
chr1:60042576–60042951	Carf-Wdr12	−1.89±0.74	−2.96±0.43	2/3	HCP	U
chr11:116347916–116348201	Sphk1	−2.63±0.39	−3.80±0.63	2/3	HCP	U
chr4:135557672–135558059	Zfp46	−2.72±0.23	−3.65±0.55	2/3	ICP	U
chr9:65857792–65858101	Ppib	−2.90±0.95	−4.16±1.23	3/3	HCP	ND

**GEN**, genistein; **DMR**, differentially methylated region.

**Position:** GEN-mediated DMRs were identified by MspI fragment-based DNA methylation typing; these DMRs contain more than two consecutive probes with log_2_ (HpaII^r^/McrBC^r^) values unidirectionally different between the control and GEN-treated samples; the difference is greater than mean ± 3SD (Figure S6). Positions of each DMR in the mouse genome assembly, February 2006, NCBI36/mm8, are shown.

**Gene(s):** the names of the gene(s) adjacent to the DMR are given. In some cases, a DMR contained two distinct, bi-directional promoters.

**Control and GEN**: the mean and standard deviation of the log_2_ (HpaII^r^/McrBC^r^) values for all probes within the DMR. Data were sorted from high to low for the Control log_2_ (HpaII^r^/McrBC^r^) data.

**DM/T**: the ratio of the number of differentially methylated (DM) probes (the difference is greater than mean ± 3SD) to the total (T) number of probes within a DMR.

**Class**: promoter classification based on the CpG density. High CpG-density promoters (HCPs) contain at least one 500-bp region with GC content >0.55 and have a ratio of observed to expected CpGs >0.6. Low CpG-density promoters (LCPs) lack the 500-bp GC-rich interval and the ratio of observed to expected CpGs is >0.4. Intermediate CpG-density promoters (ICPs) have characteristics between HCPs and LCPs.

**Ref.:** the methylation levels of the various types of tissues of the corresponding region in human genome. Methylation levels of Methylated (M), Intermediately methylated (I), and Unmethylated (U) derive from the Ensembl genome-wide DNA methylation resource (E-TABM-445). ND denotes a lack of available information about the methylation level of the human counterpart.

*This DMR integrated two proximate probes that were similarly hypomethylated following GEN treatment; one probe resided in the MspI interval of chr14:101869393–101869526, and the other was in chr14:101869523–101869657.

**Table 3 pone-0019278-t003:** Methylation levels of 16 DMRs in first exons that decreased following GEN exposure.

Position	Gene(s)	Control	GEN	DM/T	Class	Ref.
chr10:77446495–77446745	Aire	1.10±0.13	−1.09±0.27	2/2	HCP	I
chr2:152723597–152723825	Xkr7	−0.30±0.17	−2.35±0.37	2/2	HCP	U
chr16:85691690–85692049	Adamts1	−0.39±0.32	−1.76±0.49	3/4	HCP	U
chr4:138346306–138346788	Htr6	−0.40±0.36	−1.54±0.24	3/4	HCP	I
chr7:127166953–127167145	Zfp747	−0.47±0.33	−2.34±0.01	2/2	HCP	U
chr5:123655740–123655973	Mlxip	−0.56±0.11	−2.23±0.64	2/2	HCP	U
chr6:115818559–115819049	Mbd4	−0.71±0.36	−2.06±0.73	4/5	HCP	U
chr19:46185243–46185579	Elovl3	−0.73±0.33	−2.23±0.25	3/3	ICP	U
chr18:59300968–59301135	Chsy3	−1.01±0.08	−2.80±0.04	2/2	HCP	U
chr14:54635513–54635699	Tinf2	−1.12±0.20	−2.95±0.53	2/2	HCP	U
chr18:86847315–86847672	Cbln2	−1.26±0.49	−3.04±0.62	2/2	HCP	U
chr2:148098766–148098943	Thbd	−1.34±0.07	−2.63±0.01	2/2	HCP	U
chr6:120330010–120330251	Kdm5a	−1.34±0.42	−2.83±0.68	2/2	HCP	U
chr7:24007929–24008187	Zfp61	−1.35±0.18	−2.89±0.89	2/2	HCP	ND
chr16:32339489–32339720	Tctex1d2	−1.48±0.59	−3.18±0.65	2/2	HCP	U
chr5:75357283–75357527**	Gsx2	−1.96±0.06	−3.68±0.20	2/2	HCP	U

***ibid***.

**This DMR integrated two proximate probes that were similarly hypomethylated following GEN treatment; one probe resided in the MspI interval of chr5:75357283–75357391, and the other probe resided in chr5:75357388–75357527.

**Table 4 pone-0019278-t004:** Methylation levels of 19 DMRs in promoters that increased following GEN exposure.

Position	Gene(s)	Control	GEN	DM/T	Class	Ref.
chr13:114784255–114784514	Hspb3	−0.20±0.11	1.40±0.41	2/2	ICP	I
chr7:37276642–37276850	Zfp536	−0.27±0.29	0.94±0.31	2/2	HCP	U
chr11:95658180–95658391	Abi3-Gngt2	−0.42±0.07	1.16±0.13	2/2	LCP	M
chr15:74548037–74548493	4930572J05Rik	−0.99±0.33	0.04±0.40	2/4	HCP	U
chr14:119410569–119410820	Mbnl2	−1.62±0.55	−0.09±0.02	2/2	ICP	U
chr9:106679759–106679960	Vprbp	−1.69±0.28	−0.43±0.09	2/2	HCP	U
chr5:114705063–114705316	Mmab-Mvk	−1.82±0.01	−0.29±0.09	2/2	HCP	U
chr16:92355095–92355340	Rcan1	−1.83±0.30	0.02±0.08	2/2	HCP	U
chr17:34172243–34172480	Notch4	−1.83±0.57	−0.20±0.46	2/2	LCP	ND
chr3:5559307–5559483	Pxmp3	−2.00±0.25	−0.53±0.07	2/2	HCP	U
chr7:28088184–28088486	Zfp36	−2.12±0.84	−0.35±0.30	2/3	HCP	U
chr3:36743554–36744043	Exosc9	−2.14±0.89	−1.03±0.75	4/5	HCP	ND
chr2:168281700–168281915	Nfatc2	−2.24±0.50	−0.61±0.47	2/2	HCP	U
chr2:119238582–119238990	Exd1-1500003O03Rik	−2.37±0.32	−0.81±0.29	2/2	HCP	U
chr19:8837816–8838035	Tmem223-Nxf1	−2.39±0.52	0.31±0.16	2/2	HCP	U
chr7:99357657–99357927	Rps3	−2.58±0.81	−0.93±0.23	2/3	HCP	U
chr1:45870162–45870424	Slc40a1	−2.68±0.17	−1.22±0.43	2/2	ICP	U
chr11:99961777–99962052	Krt19	−3.50±0.59	−1.33±0.33	2/2	HCP	U
chr8:46831926–46832115	Cyp4v3	−3.55±0.47	−1.41±0.49	2/2	HCP	U

***ibid***.

**Table 5:** Methylation levels of 7 DMRs in first exons that increased following GEN exposure.

Position	Gene(s)	Control	GEN	DM/T	Class	Ref.
chr14:54742660–54742881	Ripk3	−1.58±0.14	−0.19±0.16	2/2	ICP	I
chr2:156412702–156413107	Dlgap4	−2.05±0.60	−0.95±0.17	2/4	HCP	I
chr14:43910711–43910948	Ptger2	−2.21±0.37	−0.84±0.36	2/2	HCP	U
chr5:45957763–45957961	1600023N17Rik	−2.23±0.22	−0.61±0.13	2/2	HCP	U
chr10:80105916–80106136	9030607L17Rik	−2.75±1.07	−0.78±0.54	2/2	HCP	U
chr7:25336561–25336903	B3GNT8	−2.82±0.29	−1.07±1.42	2/2	ICP	M
chr5:137970985–137971285	6430598A04Rik	−3.03±0.53	−1.26±0.39	3/3	HCP	I

***ibid***.

### The *Ucp1* promoter is differentially methylated upon genistein treatment

**Figure 2:**
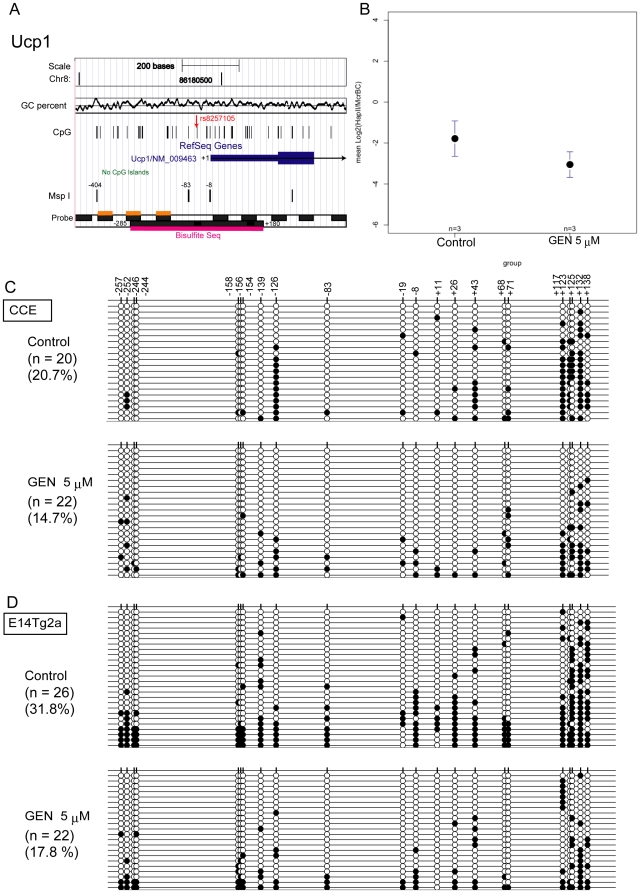
Methylation levels of the *Ucp1* promoter decreased following genistein (GEN) treatment. **A**: The genomic location and other features of the mouse *Ucp1* promoter are shown. The sequence identifier containing the *Ucp1* promoter region covers chr8: 86179166–86180966 (NCBI36/mm8 assembly), including a single nucleotide polymorphism, rs8257105, that is A/A in 129 and G/G in C57BL/6. In embryonic stem cell lines CCE and E14Tg2a with rs8257107 (A/A), the CpG and MspI recognition sequences are lost. The MspI fragment at chr8: 86180062–86180386 (from −404 to −83 relative to TSS) was identified as a GEN-mediated differentially methylated region (DMR) by MspI fragment-based DNA methylation typing. There are three NimbleGen probes (orange rectangles) in the DMR; the region from −285 to +180 (magenta rectangle) was analyzed by bisulfite sequencing. **B**: The means and standard deviations for the log_2_ (HpaII^r^/McrBC^r^) values of the three probes within the DMR (−404 to −83) are shown; the values from the GEN-treated samples (−3.05±0.47) were significantly lower than in the control samples (−1.78±0.66; p<0.0079, Student's t test). **C and D**: Bisulfite sequencing of CpGs was carried out for day 10 differentiation samples from CCE and E14Tg2a embryonic stem cell lines to compare methylation levels between control cells and cells treated with 5 µM GEN. Black and white circles indicate methylated and unmethylated CpGs, respectively.

From the genistein-mediated DMRs, we selected the *Ucp1* and *Sytl1* promoter regions as representative of the ICP and LCP promoters, respectively, for further analysis. First, we confirmed the MFMT results on the *Ucp1* gene by bisulfite sequencing (Figure 2). We identified the MspI fragment at chr8: 86180062–86180386 (NCBI36/mm8; from −404 to −83 relative to the TSS) as a genistein-mediated DMR because the mean log_2_ (HpaII^r^/McrBC^r^) value in the genistein-treated sample (−3.05±0.47) was significantly lower than in the control sample (−1.78±0.66) (Student's t test, p<0.0079) (Table 2, Figure 2A, B). Bisulfite sequencing analysis from two independent experiments revealed that methylation levels in the *Ucp1* region (−285 to +180) were significantly lower following genistein treatment (Figure 2C; Fisher's exact test, p<0.0192); the region analyzed by bisulfite sequencing overlaps with the DMR identified by the MFMT assay. Although genistein did not affect the CpG methylation level at the −83 MspI site, the methylation level of the McrBC site at −126 changed considerably (Figure 2C). Therefore, bisulfite sequencing was consistent with the MFMT assay, indicating that genistein treatment decreases the methylation levels of the *Ucp1* promoter. This decrease was also confirmed using another ES cell line, E14Tg2a (Figure 2D).

### *De novo* methylation of the *Ucp1* promoter

**Figure 3:**
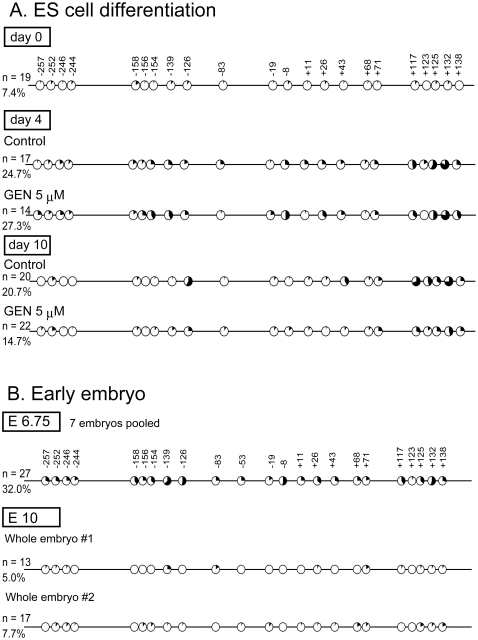
Developmental dynamics of *Ucp1* methylation and the influence of genistein (GEN). Methylation levels were analyzed by bisulfite sequencing. The black inlay is the mean methylation level of each CpG, and the left panel contains the number of sequenced clones and the mean methylation level of all CpGs. **A**: The methylation levels of the *Ucp1* promoter dynamically changed during embryonic stem (ES) cell differentiation. In undifferentiated (day 0) ES cells, most CpGs in the *Ucp1* promoter were unmethylated. Although the methylation levels increased by day 4, there was no significant difference between methylation levels in control (24.7%) and GEN-treated samples (27.3%; p<0.43, Fisher's exact test). On day 10, the methylation level of the *Ucp1* promoter in GEN-treated cells (14.7%) was significantly lower than in control cells (20.7%; p<0.0192), and the overall methylation levels were decreased on day 10 compared with day 4. **B**: The methylation levels of the *Ucp1* promoter in early embryos are shown. As the embryo developed, the methylation level declined between E6.75 and E10.

Next, we asked whether the *Ucp1* promoter region underwent *de novo* DNA methylation during ES cell differentiation. Methylation levels were analyzed by bisulfite sequencing. The Ucp1 promoter region was unmethylated before differentiation (day 0); the methylation levels increased until day 4 and decreased thereafter, and there was no significant difference in the methylation level of the *Ucp1* promoter between control and genistein-treated cells on day 4 (Fisher's exact test, p<0.43) (Figure 3A). While the developmental stage of day 4 ES cell differentiation corresponds to the gastrula stage of the early embryo [13], the day 10 ES cell differentiation stage possibly corresponds to E8-10 embryos, which are initiating early organogenesis. We observed that the methylation levels of the *Ucp1* promoter in early normal embryos were much higher at E6.75 (gastrula) than at E10 (early organogenesis) (Figure 3B), suggesting that the methylation level of the *Ucp1* promoter is transiently elevated by the time of the gastrulation stage, but is subsequently demethylated in the course of further development.

### The *Sytl1* promoter region is differentially methylated following genistein treatment

**Figure 4:**
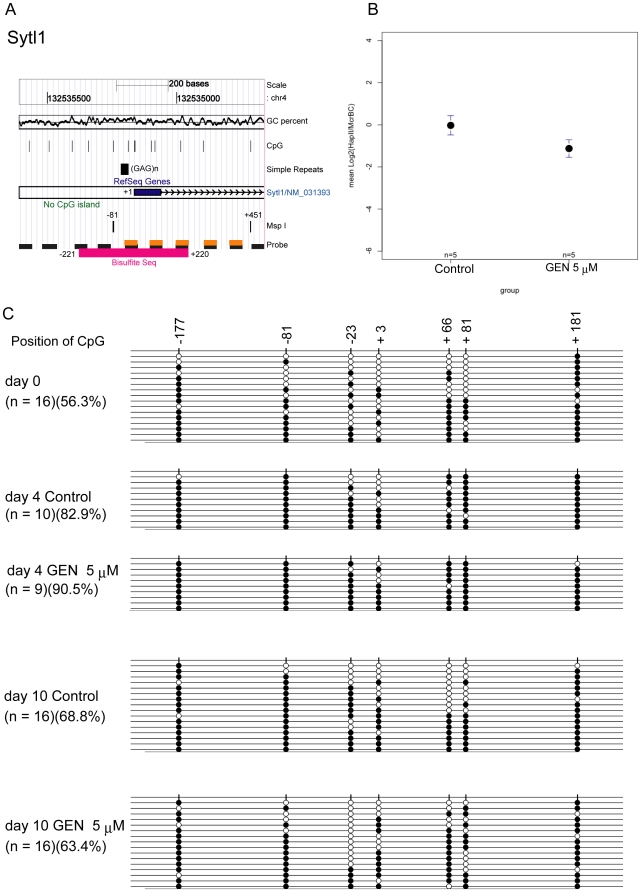
The methylation levels of the *Sytl1* promoter decreased following genistein (GEN) treatment. **A**: The genomic location and other features of the mouse *Sytl1* promoter are shown. The sequence identifier containing the *Sytl1* promoter covers chr4: 132534642–132536442 (NCBI36/mm8 assembly). MspI fragment-based DNA methylation typing identified the MspI fragment at chr4: 132534712–132535246 (−81 to +451 relative to TSS) as a GEN-mediated differentially methylated region (DMR). The simple repeat (GAG)n is found in the region (from −51 to −24). Five probes reside in the DMR (orange rectangles); the region from −221 to +220 (magenta rectangle) was analyzed with bisulfite sequencing. **B**: The mean and standard deviations for the log_2_ (HpaII^r^/McrBC^r^) values of the five probes within the DMR (−81 to +451) are shown; the value in the sample treated with 5 µM GEN (−1.13±0.44) was lower than that in the control sample (−0.03±0.48; p<0.027, Student's t test). **C**: Bisulfite sequencing of CpGs was carried out for day 0, day 4, and day 10 of embryonic stem cell differentiation samples to monitor the effect of GEN treatment.

We also used bisulfite sequencing to investigate the methylation states of another genistein-mediated DMR, the *Sytl1* promoter (Figure 4). We identified the MspI fragment at chr4: 132534712–132535246 (−81 to +451 relative to the TSS) as a genistein-mediated DMR because the mean log_2_ (HpaII^r^/McrBC^r^) value in the MFMT analysis of the genistein-treated samples (−1.13±0.44) was significantly lower than in the control samples (−0.03±0.48) (Student's t test, p<0.027)(Table 2, Figures 4A, B). Bisulfite sequencing of the region from −221 to +220 (7 CpGs; Figure 4C) in day 0, day 4, and day 10 CCE ES differentiation samples revealed that the *Sytl1* promoter was moderately methylated on day 0. The methylation level increased until day 4 in a manner that genistein treatment did not affect. The methylation level had decreased by day 10, but of note, the methylation decrease occurred at different positions in the control (+66) and genistein-treated (−23) cells. The overall methylation level following genistein treatment was lower than in control cells on day 10. The CpG methylation level at the HpaII site at −81 was also slightly lower in genistein-treated cells than in control cells, suggesting that the MFMT assay could detect the slight difference in methylation levels of this HpaII site. Thus, genistein treatment influenced the methylation levels of the *Ucp1* and *Sytl1* promoters in a similar fashion.

## Discussion

Epidemiological and animal studies have demonstrated that nutrition and other environmental stimuli influence prenatal developmental pathways, thereby inducing permanent changes in metabolism and chronic disease susceptibility. Epigenetic alteration caused by the intrauterine environment may partly explain trait diversity, including disease phenotypes. Nevertheless, little is known about the causal relationship between the intrauterine environment and epigenetic dysregulation, probably because somatic methylome establishment consists of multiple developmental steps. Considerable attention has been paid to alterations in the fetal epigenome induced by nutritional stimuli *in utero*, as clearly demonstrated by changes in the methylation levels of the *A^vy^* allele resulting from maternal intake of genistein [1], bisphenol A [14], or methyl donors [15], [16]. These somatic epigenetic changes are thought to have occurred before germ layer differentiation in the early embryo [3]. Therefore, it is important to elucidate the effects of environmental modification on the fetal epigenome, particularly in this early developmental stage.

Because the *in vitro* ES cell differentiation by means of embryoid body formation is widely used for analysis of early embryogenesis around the post-implantation period, we compared the changes in DNA methylation states of the differentiated ES cell cultures between control and genistein-supplemented conditions. We added genistein to ES cell cultures at a physiological concentration of 5 µM without impairing the overall differentiation for at least 10 days (Figures S1 and S2). As expected, genistein treatment did not affect global DNA methylation patterns (Figure 1), but we could identify 74 regions proximal to TSSs that were differentially methylated following genistein treatment (Tables 2–5). We performed gene ontology analysis using the DAVID Functional Annotation tool for DMRs; however, a specific category was not significantly enriched in the genistein-mediated DMRs. This result may be attributable to an effect of genistein on a general or unspecified pathway in the early embryonic stage rather than on differentiated functions.

We also investigated in detail the methylation states of the *Ucp1* and *Sytl1* promoters (Figures 2–3 and 4, respectively). The initiation time for *de novo* methylation varies from one promoter to another, and some promoter methylation is erased during somatic development [5]. Similarly, we found that the methylation levels of the *Ucp1* and *Sytl1* promoters dynamically change within a short period of early embryonic development. *De novo* methylation and subsequent demethylation starts early for these promoters. Methylation of the *Ucp1* promoter substantially decreased during the E6. 75-10 stage of the embryo. Similarly, the *Ucp1* and *Sytl1* promoters underwent *de novo* DNA methylation until day 4 of ES differentiation, and were subsequently demethylated by day 10 (Figures 3 and 4). It was interesting that genistein treatment did not affect *de novo* methylation, as peak methylation levels did not differ between control and genistein samples; in contrast, genistein treatment changed methylation levels in the later stage (Figures 3 and 4). Because early tissue precursor cells appear after gastrulation, it is tempting to speculate that genistein changes the methylation prototype of those precursor cells. The increase in the expression level of ERα (*Esr1*) could partly explain the genistein sensitivity on day 10 observed here (Figure S8), but it remains to be further elucidated whether nuclear receptors such as ERα and/or ERβ are involved in alteration of DNA methylation levels in genistein-mediated DMRs.

Ucp1 functions as a mitochondrial uncoupling protein in brown adipose tissue; the CpG methylation level in the Ucp1 enhancer (especially at the CRE3 site) is remarkably low in brown adipose tissue cells [17]. On the other hand, promoter methylation is known to be important for Ucp1 silencing in white adipose tissue [17], [18]. In this study, upon ES differentiation, the Ucp1 enhancer gained methylation, and genistein treatment rather amplified this increase in methylation levels (Figure S9). Thus, the genistein-mediated decrease in methylation levels was restricted only to the promoter region. Mitochondrial biosynthesis is activated upon cell-fate commitment during the course of ES cell differentiation [19]. In addition, genistein and other isoflavones promote mitochondrial biogenesis [20]. These issues could be relevant to the regulation of Ucp1 methylation states and warrant further studies.

Sytl1 functions as a Rab27 effector protein in a wide variety of cells [21], [22], [23]. Genistein treatment also altered the methylation patterns of the *Sytl1* promoter (Figure 4). In contrast to the *Ucp1* promoter, the decrease in methylation following genistein treatment occurred preferentially at the −23 CpG site. To study the somatic methylation pattern of the *Sytl1* promoter, DNA was extracted from adult mouse liver and lung and analyzed (Figure S10). The methylation patterns characteristic of hypomethylation at CpG −23 were observed in the genistein-exposed ES cells on day 10 as well as in adult somatic tissues. Again, genistein treatment lowered the methylation level of the *Sytl1* promoter, which could lead to the formation of a somatic DNA methylation pattern.

It is unclear whether the genistein-mediated differential methylation detected at the early embryonic stage in intrinsic genes (*Ucp1* and *Sytl1*) persists until later stages of development. Our demonstration that the methylation levels changed following genistein exposure during the post gastrulation period is in accord with the idea that the genistein-mediated methylation changes in *A^vy^* are generated before germ layer differentiation. The epigenetic changes established in tissue stem cells that start to appear at the post-gastrulation period may persist until the later stages of development, or they may be modified during terminal differentiation of individual tissues. The methylation dynamics at sequential stages of development must be further clarified by spatiotemporal investigations.

We compared our genistein-mediated DMRs with the DMRs previously found in intrauterine growth retardation (IUGR) neonates [24]. There were no overlaps in the lists for top genes between genistein-mediated DMRs and IUGR DMRs. However, both types of DMRs seemed to share important similarities. First of all, the magnitude of DNA methylation changes for both types of DMRs was markedly less than many tissue-specific or cancer-related differences. In addition, long-range changes in methylation levels did not occur over a gene locus in both DMRs. Rather, the methylation changes specifically occurred at particular sites among multiple alternative promoters in some genes. As for *Ucp1* and *Sytl1*, they do not seem to have alternative promoters, yet the promoter methylation levels also were changed in the IUGR neonates, albeit in the opposite direction of genistein treatment (higher methylation in IUGR than in control samples) [24]. It would be interesting to investigate whether these two genes are commonly sensitive to intrauterine environmental factors.

To the best of our knowledge, this study is the first report of the effects of genistein treatment on DNA methylation patterns during ES cell differentiation. We demonstrated that genistein perturbed methylation regulation subsequent to *de novo* methylation, resulting in alteration of the methylation levels of a subset of promoters in an early embryonic stage. In the future, we plan to investigate how this alteration influences lifetime methylation patterns.

## Supporting Information

Figure S1 **Treatment with 5 µM genistein (GEN) does not impede cardiomyocyte differentiation of embryonic stem cells.** Five culture conditions were tested for cardiomyocyte differentiation: no additives, dimethyl sulfoxide (DMSO) solvent only, 1 µM GEN, 5 µM GEN, and 10 µM GEN. Embryoid body (EB) formation was induced by hanging-drop culture (one drop contains 20 µl cell suspension at 4×10^4^ cells/ml) without leukemia inhibitory factor for two days, followed by two days of suspension culture. The EBs were transferred to a 100-mm gelatinized dish under individual culture conditions. Differentiation was determined by microscopic inspection of 20 EB outgrowths for each plate. The number of EBs containing contracting cardiomyocytes was counted every day; GEN exposure did not inhibit cardiomyocyte differentiation up to concentrations of 5 µM.
(TIF)

Figure S2 **Marker gene expressions during embryonic stem cell differentiation.** The expression levels of differentiation marker genes were analyzed by real-time PCR assay. Regardless of genistein treatment, the decline of *Pou5f1* (a pluripotency marker) and the transient elevation of *T* (a gastrulation marker) expression occurred, indicating that normal ES cell differentiation was proceeding. The elevated expression of ectoderm markers (*Fgf5* and *Wnt3a*) was similarly observed both in control and genistein-treated cells on day 4. The expression of mesoderm (*Pdgfra*, *Tbx5*) and endoderm (*GATA4*, *GATA6*) markers was also similarly increased in both conditions on day 10.
(TIF)

Figure S3 **Schematic outlines for MspI fragment-based DNA methylation typing (MFMT) assay and NimbleGen probe selection.** For MFMT, probes were selected from the NimbleGen promoter array probe set. Probes carrying MspI sites present short lengths for annealing to the amplified PCR products; therefore, NimbleGen probes carrying MspI sequences were excluded from further analysis. Because the PCR products were 100–1000 bp long, probes were selected that were enclosed by two MspI sites separated by that size range. Genomic DNA was initially digested by MspI and ligated to an oligonucleotide adaptor pair. The ligated products were digested by a methylation-sensitive restriction enzyme (HpaII or McrBC), and the digestion-resistant fragments were amplified by PCR. If the CpG at the end of the MspI fragment was unmethylated, the adaptor-ligated product was digested by the HpaII enzyme (the red zigzag lines in the left column). If the MspI fragment contained McrBC target CpGs and those CpGs were methylated, the adaptor-ligated product was digested by the McrBC enzyme (the red zigzag lines in the right column). However, HpaII-resistant and McrBC-resistant fragments remained and were labeled with Alexa 555 or 647, respectively, and cohybridized on a mouse promoter tiling array. The log_2_ (HpaII^r^/McrBC^r^) values were calculated for selected probes for the MFMT assay.
(TIF)

Figure S4 **Validation studies of the MFMT assay using the methylation control regions.** The methylation control regions were selected based on the published data reported in [6] (see Materials and Methods). The mean log_2_ (HpaII^r^/McrBC^r^) values of control and genistein-treated samples in the methylation-positive regions are plotted with blue dots, while those in the methylation-negative regions are indicated by red dots. The gene names for all seven positive controls and the 18 negative controls with lower mean log_2_ (HpaII^r^/McrBC^r^) values are shown. While the mean log_2_ (HpaII^r^/McrBC^r^) values for methylation-positive regions are above 0, those for methylation-negative regions are below 0 both in control and genistein-treated cells.
(TIF)

Figure S5 **Probe positions and their mean log_2_ (HpaII^r^/McrBC^r^) values in the methylation-positive (hypermethylated) and –negative (hypomethylated) regions.** Two methylation-positive (*Dazl* (A), *Spo11* (B)) and two methylation-negative (*Dhfr* (C), *Psmd1* (D)) control regions, the examples in Figure S4, were analyzed. For control samples and samples treated with 5 µM genistein (GEN), the means and standard deviations for the log_2_ (HpaII^r^/McrBC^r^) values of all probes within the fragment are shown.
(TIF)

Figure S6 **Distribution of log_2_ (HpaII^r^/McrBC^r^) differences between control and genistein (GEN)-treated samples.** The log_2_ (HpaII^r^/McrBC^r^) measurement from the GEN-treated dataset was subtracted from the corresponding control value for each probe. The 1330 probes above the upper limits of the mean consecutive difference (mean plus three standard deviations; right arrow) were regarded as hypomethylated probes under GEN treatment, while the 952 probes below the lower limits of the mean consecutive difference (mean minus three standard deviations; left arrow) were hypermethylated.
(TIF)

Figure S7 **Schematic outlines of the identification of a genistein (GEN)-mediated differentially methylated region (DMR).** **A**. There is a high linear correlation between the log_2_ (HpaII^r^/McrBC^r^) values from the control and 5 µM GEN-treated samples (mean = 0.048, standard deviation = 0.379). We identified 1330 hypomethylated probes and 952 hypermethylated probes, a subset of which localized within close proximity of a given genomic position. In a model scheme, three differentially methylated probes (black rectangles) appear in a region with a unique sequence identifier (SEQ_ID), and two consecutive probes are located within the same MspI fragment. **B**. We defined a genistein (GEN)-mediated differentially methylated region (DMR) as a MspI fragment within which more than two consecutive probes show the same directional log_2_ (HpaII^r^/McrBC^r^) changes. The example of a GEN-mediated DMR in the *Notch3* promoter is shown. Consecutive probes 1, 2, and 3 were extracted from the set of 1330 hypomethylated probes; these three probes localized to the same MspI fragment in the *Notch3* promoter region. Therefore, this MspI fragment region was identified as one of the GEN-mediated DMRs (Table 2).
(TIF)

Figure S8 **Expression levels of** ***Esr1*** **and** ***Esr2*** **.** The expression levels of *Esr1* and *Esr2* genes were analyzed by real-time PCR assay. The expression of *Esr1* increased as ES cells differentiated, while the *Esr2* expression level was constantly low throughout the course of ES cell differentiation.
(TIF)

Figure S9 **Hypermethylation of the Ucp1 enhancer was not changed by genistein (GEN) treatment.** Bisulfite sequencing of CpGs in the Ucp1 enhancer region was performed for day 0 and day 10 cells either unexposed (control) or exposed to 5 µM GEN. The mean methylation level of all CpGs is shown in the left panel. The core sequences (CGTCA) of the CRE3 and CRE2 sites coincide with the CpGs at −2581 and −2540. The methylation level of the enhancer region, including the CRE sites, was higher at day 10 than at day 0, regardless of GEN treatment.
(TIF)

Figure S10 **Methylation patterns of the** ***Sytl1*** **promoter in somatic tissues in adult mice.** Bisulfite sequencing of CpGs in the *Sytl1* promoter region was carried out for liver and lung tissue cells of an adult female mouse. The analyzed region is the same as that depicted in Figure 4. The methylation level at the −23 CpG site is consistently low in both tissues.
(TIF)

Table S1 **Primers and Universal Probe Library probes for real-time PCR.** Detailed information for Universal Probe Library probes is available online at the “Universal Probe Library Assay Design Center” of Roche Applied Science.
(DOC)

Table S2 **Primers used for bisulfite sequencing.**
(DOC)

Table S3 **Gene ontology analysis of genes adjacent to or contained within differentially methylated regions.**
(DOC)
