# Supplementary figures and images for: Genome-Wide DNA Methylation Analysis Reveals Phytoestrogen Modification of Promoter Methylation Patterns during Embryonic Stem Cell Differentiation
Source: PLoS One. 2011 Apr 29;6(4):e19278. doi: 10.1371/journal.pone.0019278 (PMC3084807; doi:10.1371/journal.pone.0019278)

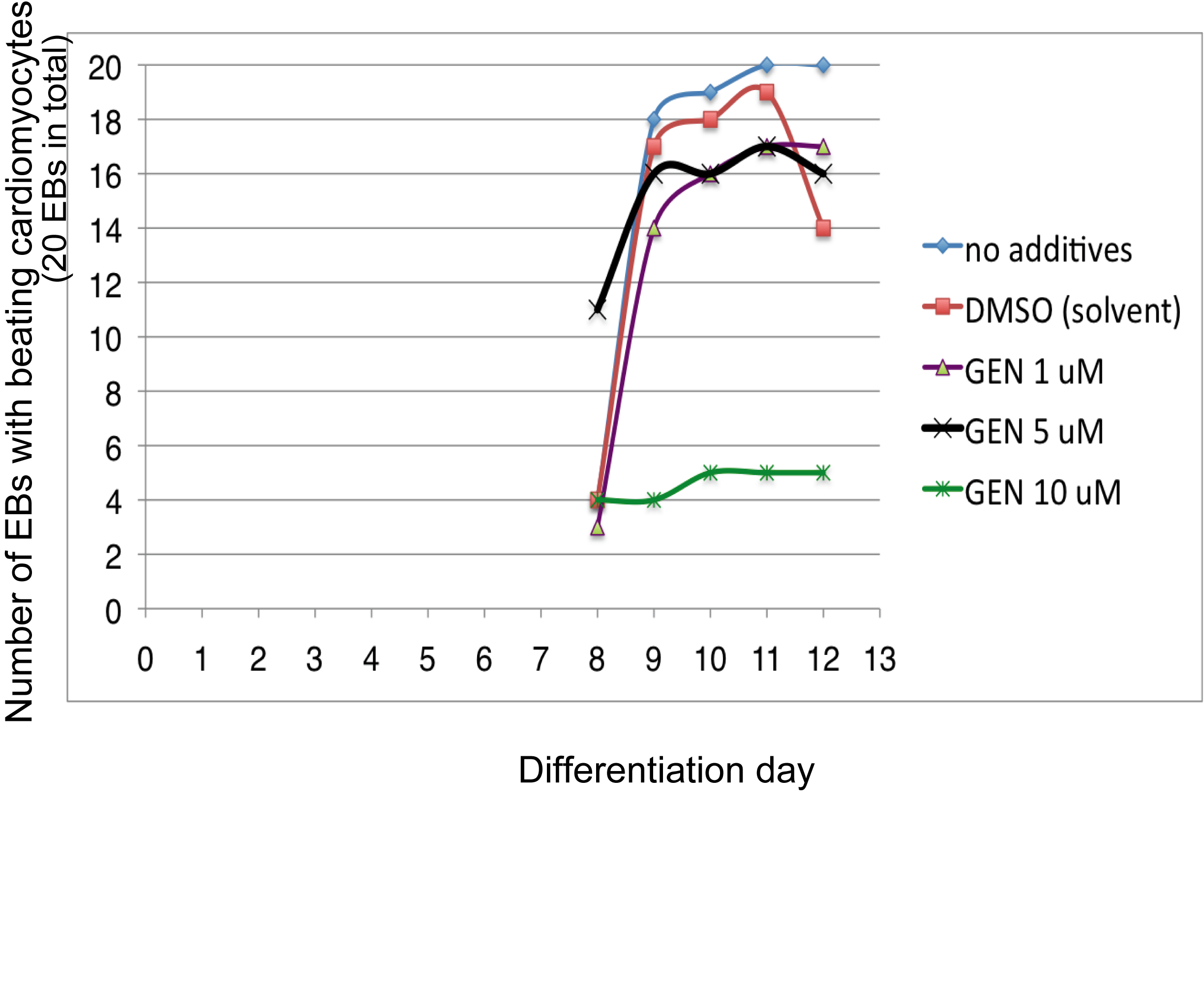

Supplement: Figure S1 — Treatment with 5 µM genistein (GEN) does not impede cardiomyocyte differentiation of embryonic stem cells. Five culture conditions were tested for cardiomyocyte differentiation: no additives, dimethyl sulfoxide (DMSO) solvent only, 1 µM GEN, 5 µM GEN, and 10 µM GEN. Embryoid body (EB) formation was induced by hanging-drop culture (one drop contains 20 µl cell suspension at 4×10^4^ cells/ml) without leukemia inhibitory factor for two days, followed by two days of suspension culture. The EBs were transferred to a 100-mm gelatinized dish under individual culture conditions. Differentiation was determined by microscopic inspection of 20 EB outgrowths for each plate. The number of EBs containing contracting cardiomyocytes was counted every day; GEN exposure did not inhibit cardiomyocyte differentiation up to concentrations of 5 µM. (TIF) [file pone.0019278.s001.tif]

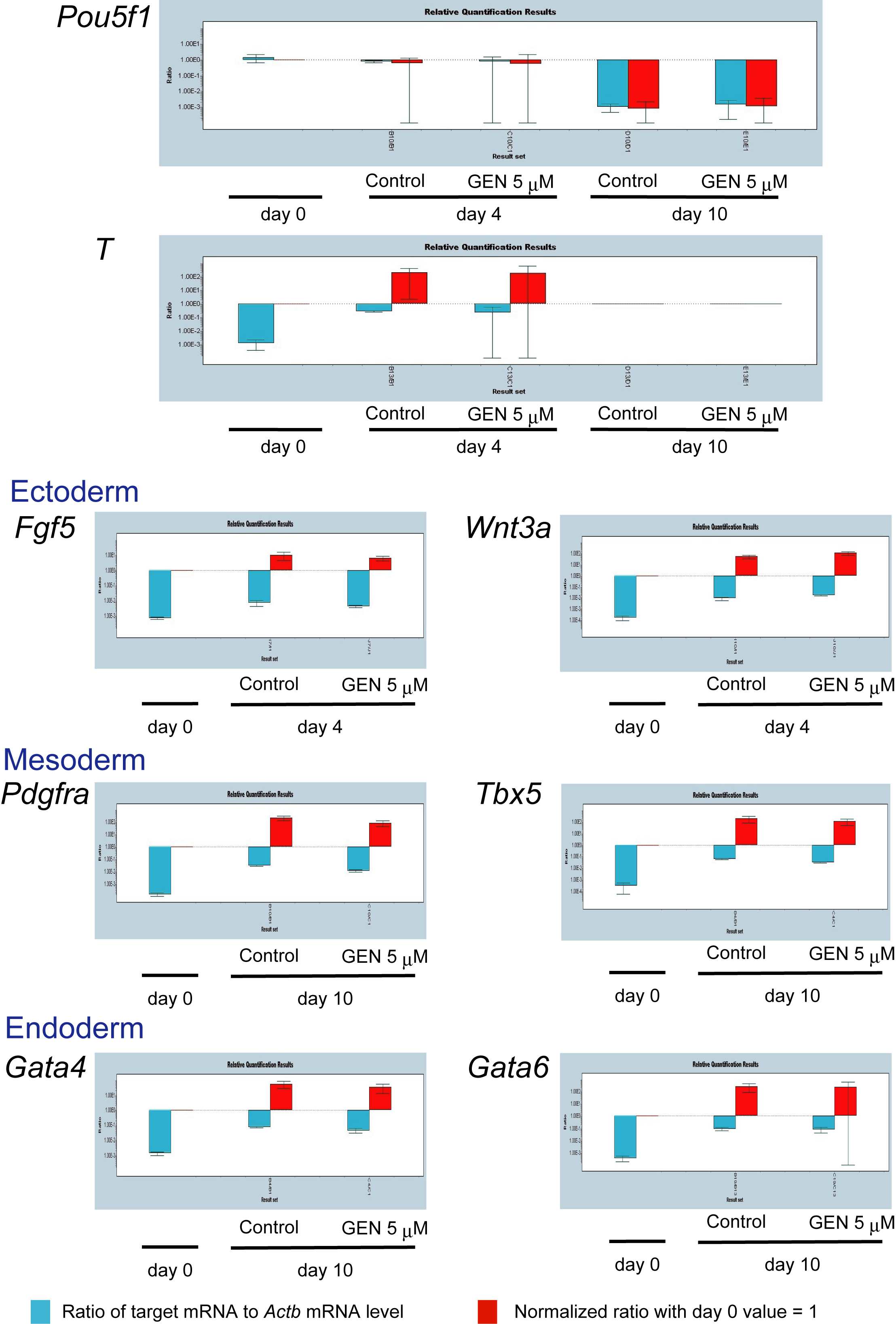

Supplement: Figure S2 — Marker gene expressions during embryonic stem cell differentiation. The expression levels of differentiation marker genes were analyzed by real-time PCR assay. Regardless of genistein treatment, the decline of Pou5f1 (a pluripotency marker) and the transient elevation of T (a gastrulation marker) expression occurred, indicating that normal ES cell differentiation was proceeding. The elevated expression of ectoderm markers (Fgf5 and Wnt3a) was similarly observed both in control and genistein-treated cells on day 4. The expression of mesoderm (Pdgfra, Tbx5) and endoderm (GATA4, GATA6) markers was also similarly increased in both conditions on day 10. (TIF) [file pone.0019278.s002.tif]

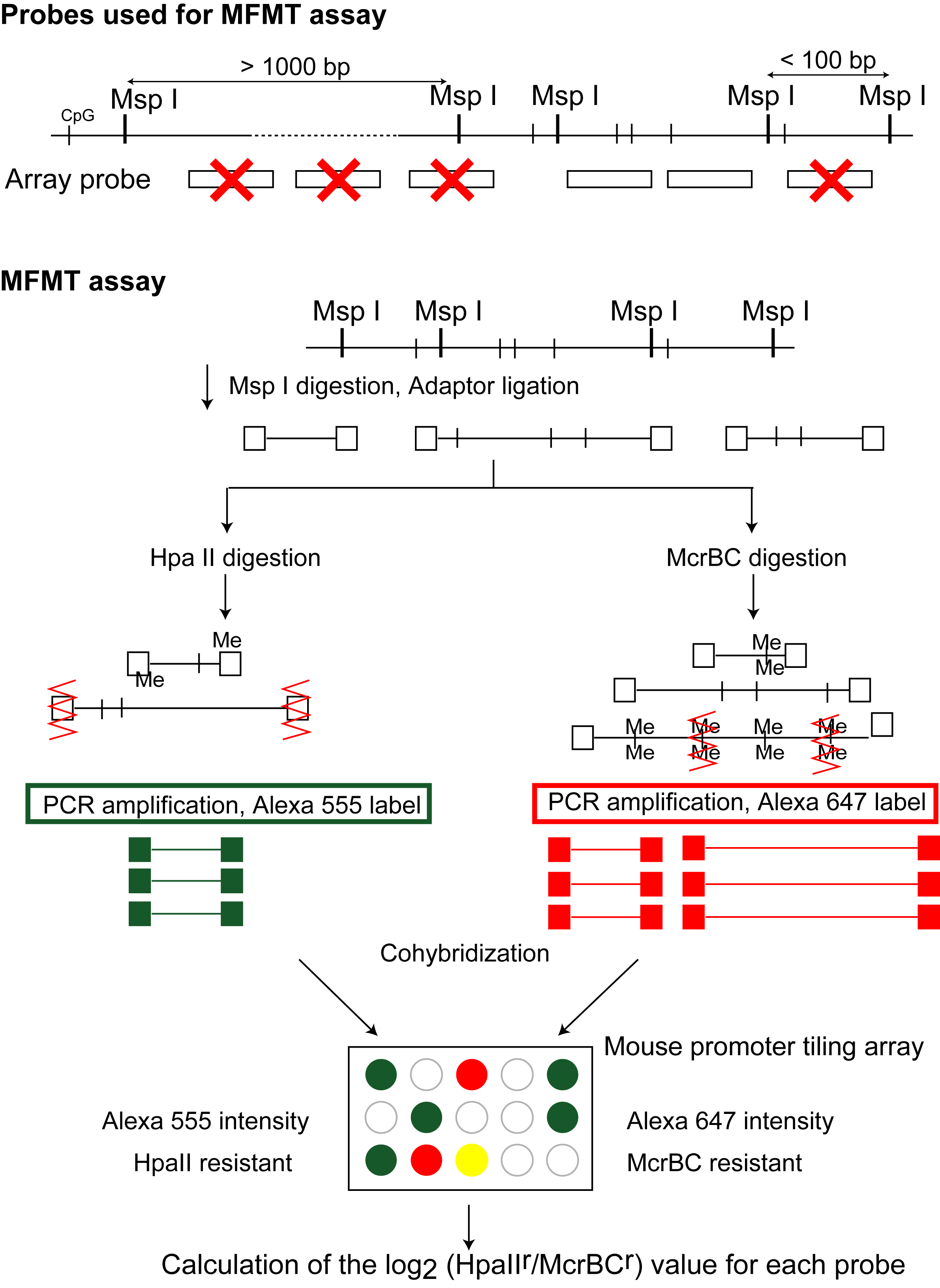

Supplement: Figure S3 — Schematic outlines for MspI fragment-based DNA methylation typing (MFMT) assay and NimbleGen probe selection. For MFMT, probes were selected from the NimbleGen promoter array probe set. Probes carrying MspI sites present short lengths for annealing to the amplified PCR products; therefore, NimbleGen probes carrying MspI sequences were excluded from further analysis. Because the PCR products were 100–1000 bp long, probes were selected that were enclosed by two MspI sites separated by that size range. Genomic DNA was initially digested by MspI and ligated to an oligonucleotide adaptor pair. The ligated products were digested by a methylation-sensitive restriction enzyme (HpaII or McrBC), and the digestion-resistant fragments were amplified by PCR. If the CpG at the end of the MspI fragment was unmethylated, the adaptor-ligated product was digested by the HpaII enzyme (the red zigzag lines in the left column). If the MspI fragment contained McrBC target CpGs and those CpGs were methylated, the adaptor-ligated product was digested by the McrBC enzyme (the red zigzag lines in the right column). However, HpaII-resistant and McrBC-resistant fragments remained and were labeled with Alexa 555 or 647, respectively, and cohybridized on a mouse promoter tiling array. The log2 (HpaIIr/McrBCr) values were calculated for selected probes for the MFMT assay. (TIF) [file pone.0019278.s003.tif]

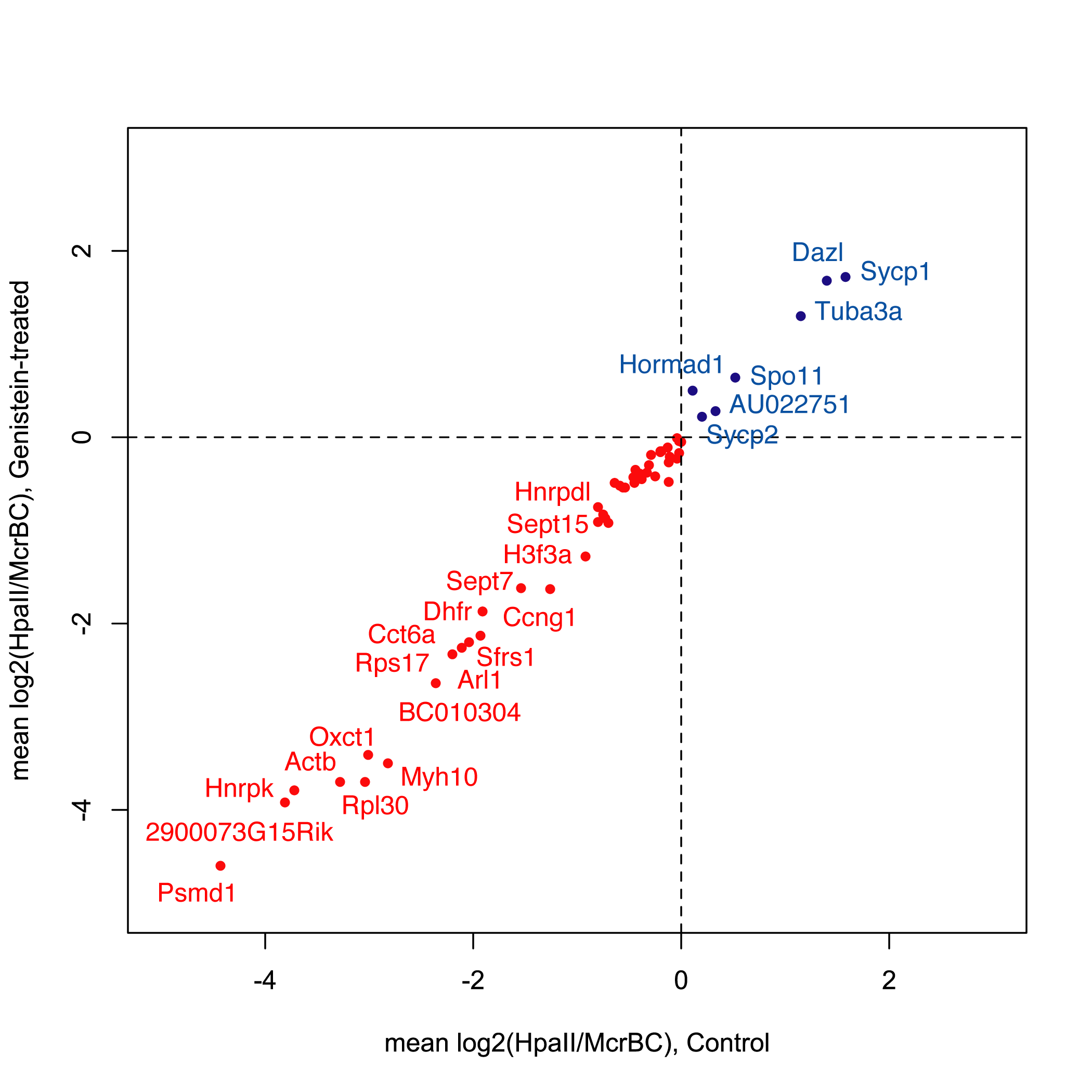

Supplement: Figure S4 — Validation studies of the MFMT assay using the methylation control regions. The methylation control regions were selected based on the published data reported in [6] (see Materials and Methods). The mean log2 (HpaIIr/McrBCr) values of control and genistein-treated samples in the methylation-positive regions are plotted with blue dots, while those in the methylation-negative regions are indicated by red dots. The gene names for all seven positive controls and the 18 negative controls with lower mean log2 (HpaIIr/McrBCr) values are shown. While the mean log2 (HpaIIr/McrBCr) values for methylation-positive regions are above 0, those for methylation-negative regions are below 0 both in control and genistein-treated cells. (TIF) [file pone.0019278.s004.tif]

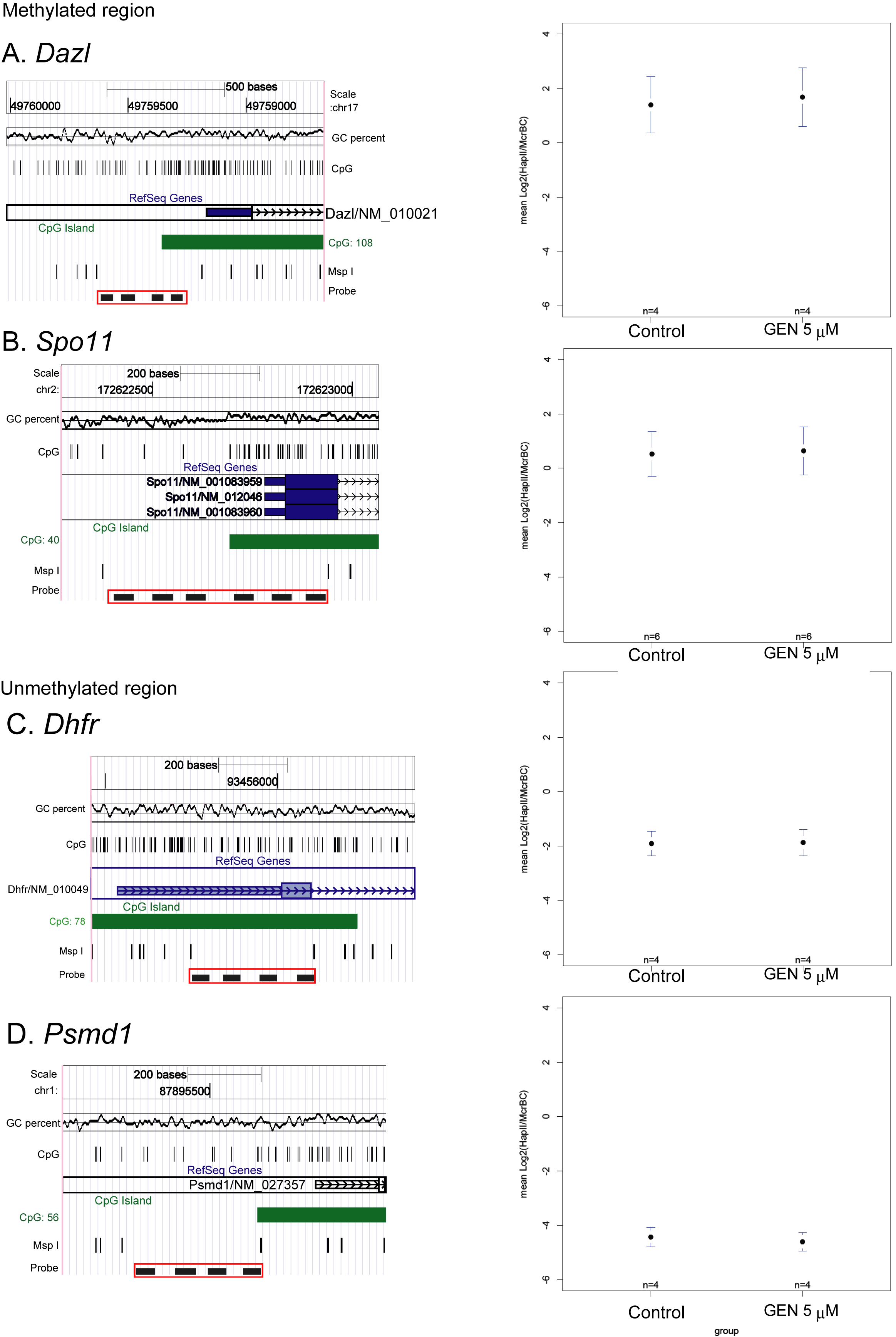

Supplement: Figure S5 — Probe positions and their mean log2 (HpaIIr/McrBCr) values in the methylation-positive (hypermethylated) and –negative (hypomethylated) regions. Two methylation-positive (Dazl (A), Spo11 (B)) and two methylation-negative (Dhfr (C), Psmd1 (D)) control regions, the examples in Figure S4, were analyzed. For control samples and samples treated with 5 µM genistein (GEN), the means and standard deviations for the log2 (HpaIIr/McrBCr) values of all probes within the fragment are shown. (TIF) [file pone.0019278.s005.tif]

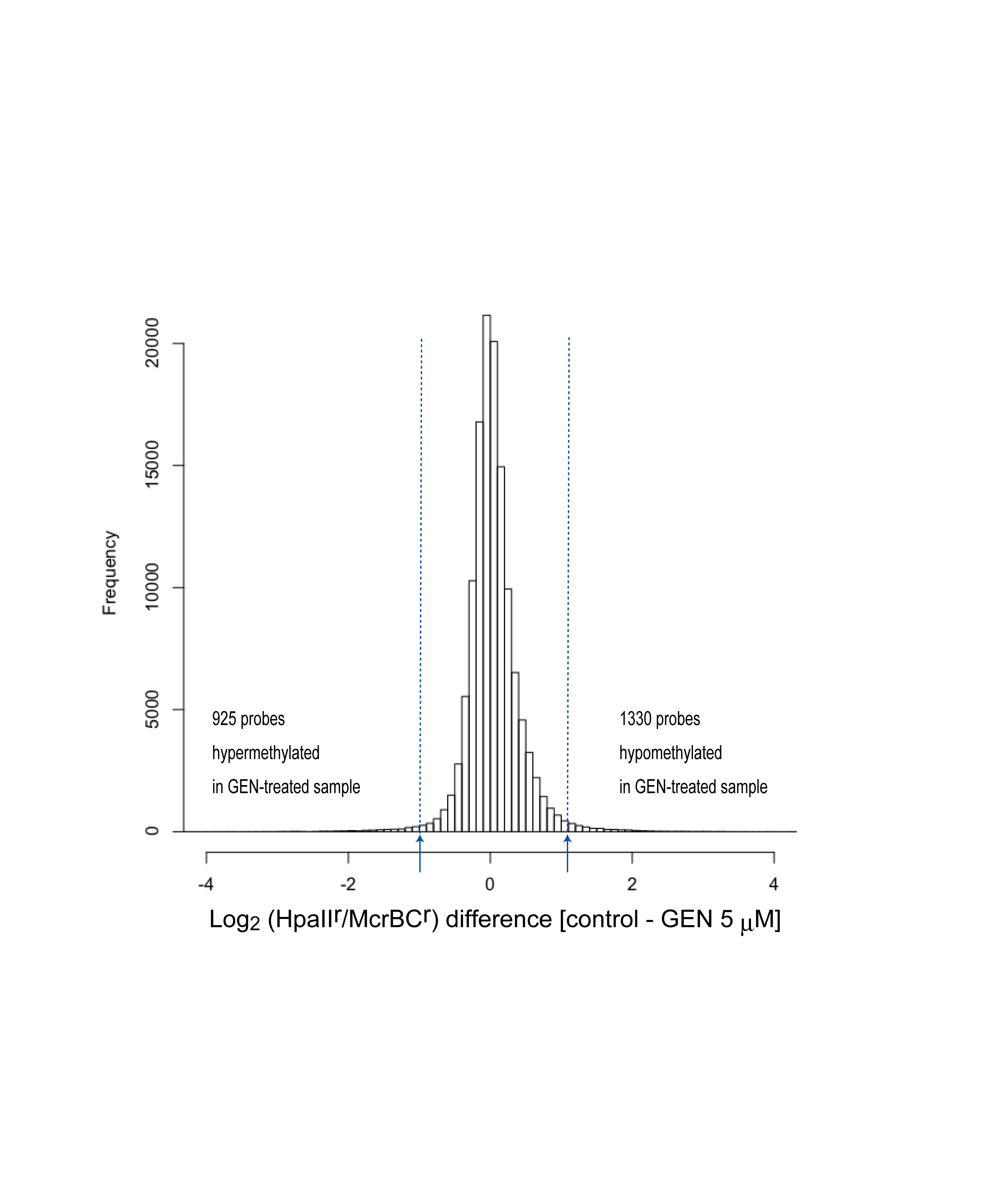

Supplement: Figure S6 — Distribution of log2 (HpaIIr/McrBCr) differences between control and genistein (GEN)-treated samples. The log2 (HpaIIr/McrBCr) measurement from the GEN-treated dataset was subtracted from the corresponding control value for each probe. The 1330 probes above the upper limits of the mean consecutive difference (mean plus three standard deviations; right arrow) were regarded as hypomethylated probes under GEN treatment, while the 952 probes below the lower limits of the mean consecutive difference (mean minus three standard deviations; left arrow) were hypermethylated. (TIF) [file pone.0019278.s006.tif]

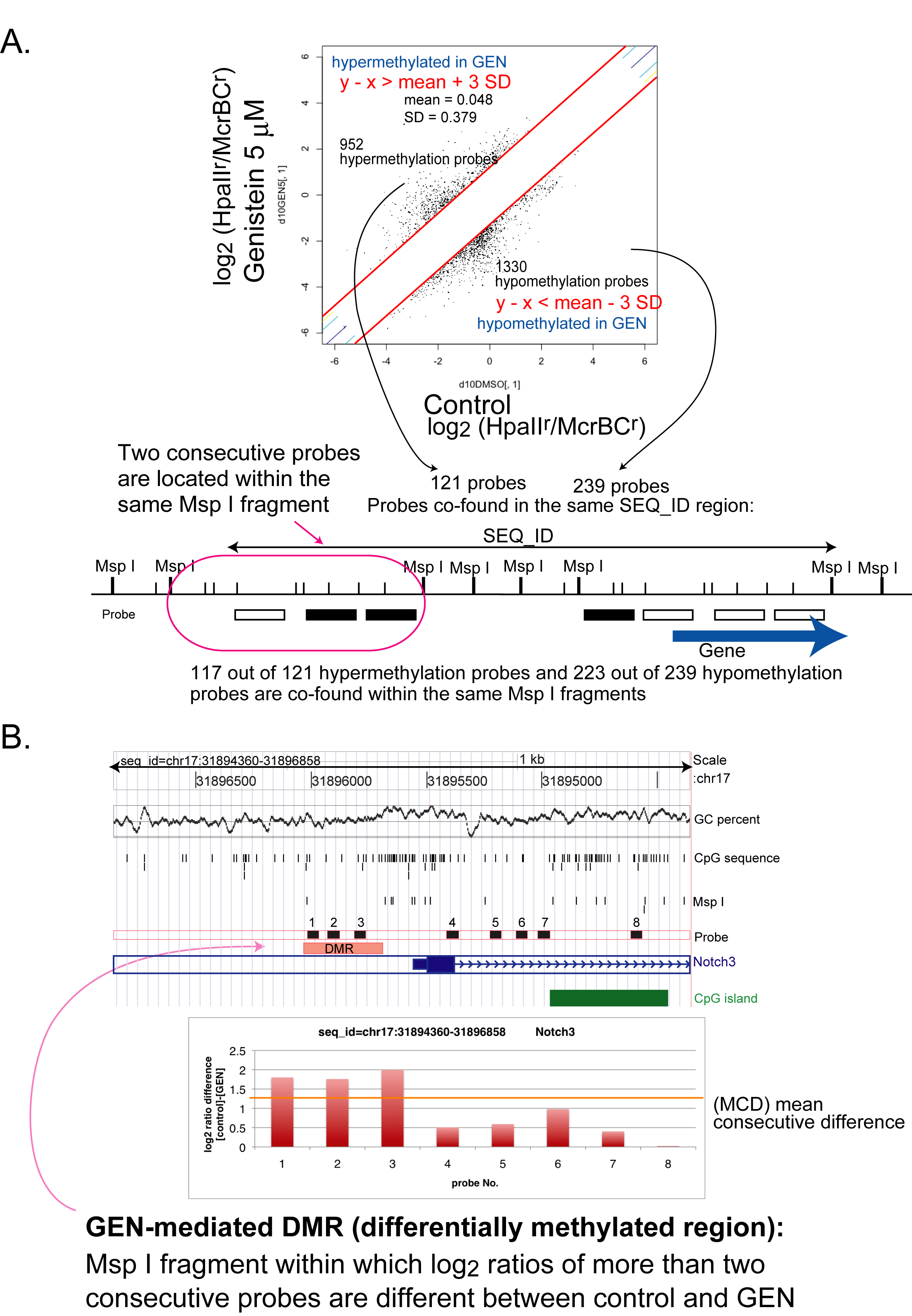

Supplement: Figure S7 — Schematic outlines of the identification of a genistein (GEN)-mediated differentially methylated region (DMR). A. There is a high linear correlation between the log2 (HpaIIr/McrBCr) values from the control and 5 µM GEN-treated samples (mean = 0.048, standard deviation = 0.379). We identified 1330 hypomethylated probes and 952 hypermethylated probes, a subset of which localized within close proximity of a given genomic position. In a model scheme, three differentially methylated probes (black rectangles) appear in a region with a unique sequence identifier (SEQ_ID), and two consecutive probes are located within the same MspI fragment. B. We defined a genistein (GEN)-mediated differentially methylated region (DMR) as a MspI fragment within which more than two consecutive probes show the same directional log2 (HpaIIr/McrBCr) changes. The example of a GEN-mediated DMR in the Notch3 promoter is shown. Consecutive probes 1, 2, and 3 were extracted from the set of 1330 hypomethylated probes; these three probes localized to the same MspI fragment in the Notch3 promoter region. Therefore, this MspI fragment region was identified as one of the GEN-mediated DMRs (Table 2). (TIF) [file pone.0019278.s007.tif]

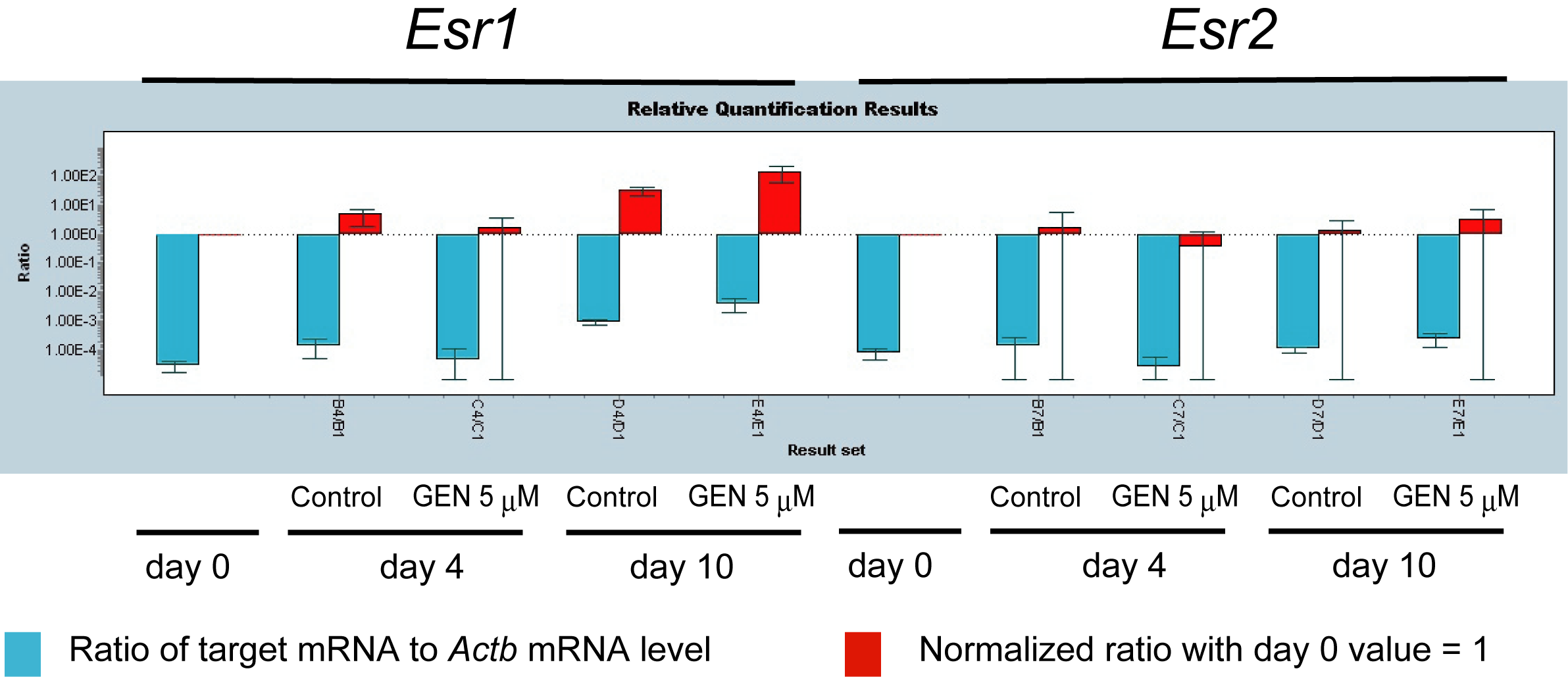

Supplement: Figure S8 — Expression levels of Esr1 and Esr2 . The expression levels of Esr1 and Esr2 genes were analyzed by real-time PCR assay. The expression of Esr1 increased as ES cells differentiated, while the Esr2 expression level was constantly low throughout the course of ES cell differentiation. (TIF) [file pone.0019278.s008.tif]

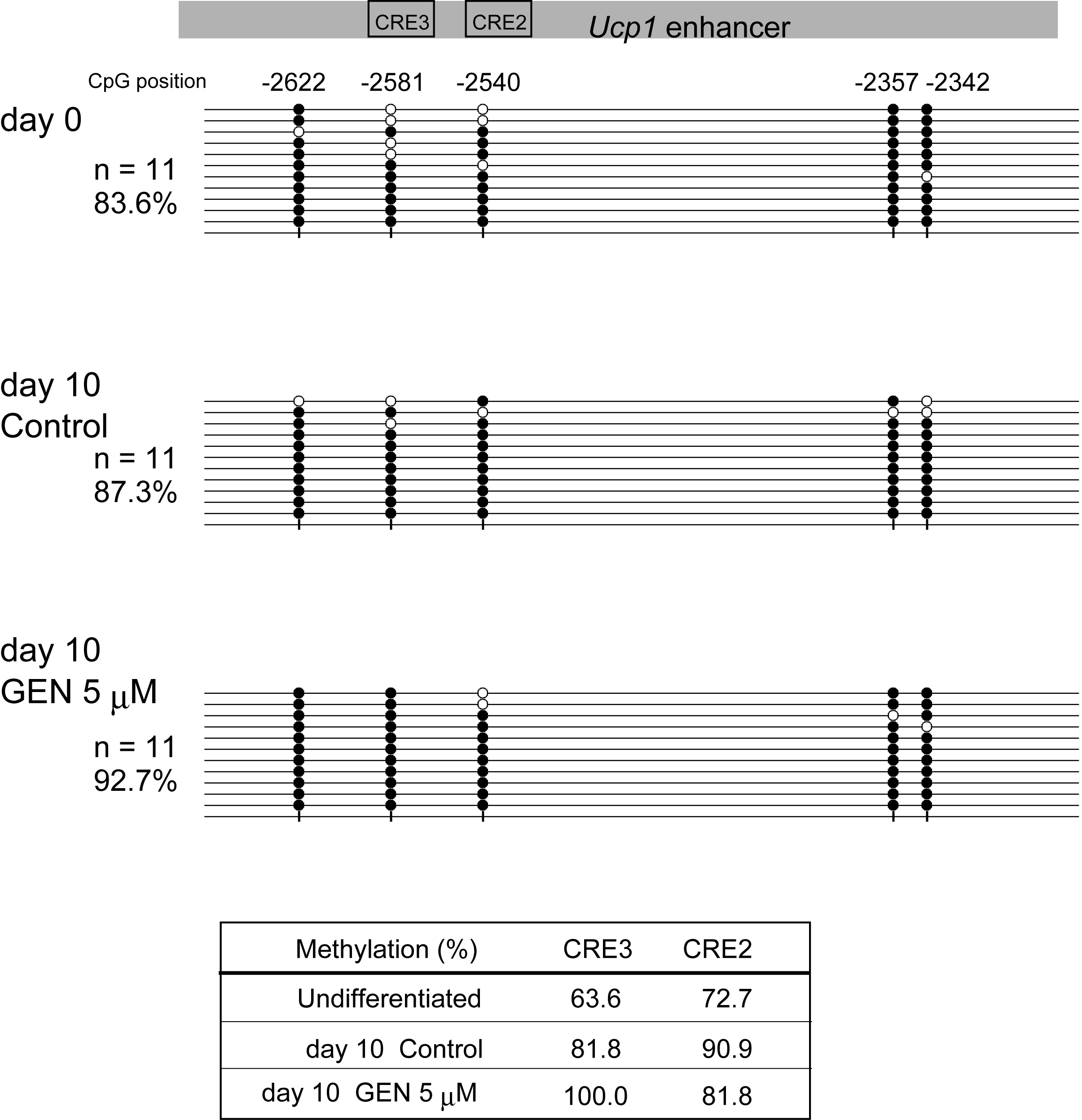

Supplement: Figure S9 — Hypermethylation of the Ucp1 enhancer was not changed by genistein (GEN) treatment. Bisulfite sequencing of CpGs in the Ucp1 enhancer region was performed for day 0 and day 10 cells either unexposed (control) or exposed to 5 µM GEN. The mean methylation level of all CpGs is shown in the left panel. The core sequences (CGTCA) of the CRE3 and CRE2 sites coincide with the CpGs at −2581 and −2540. The methylation level of the enhancer region, including the CRE sites, was higher at day 10 than at day 0, regardless of GEN treatment. (TIF) [file pone.0019278.s009.tif]

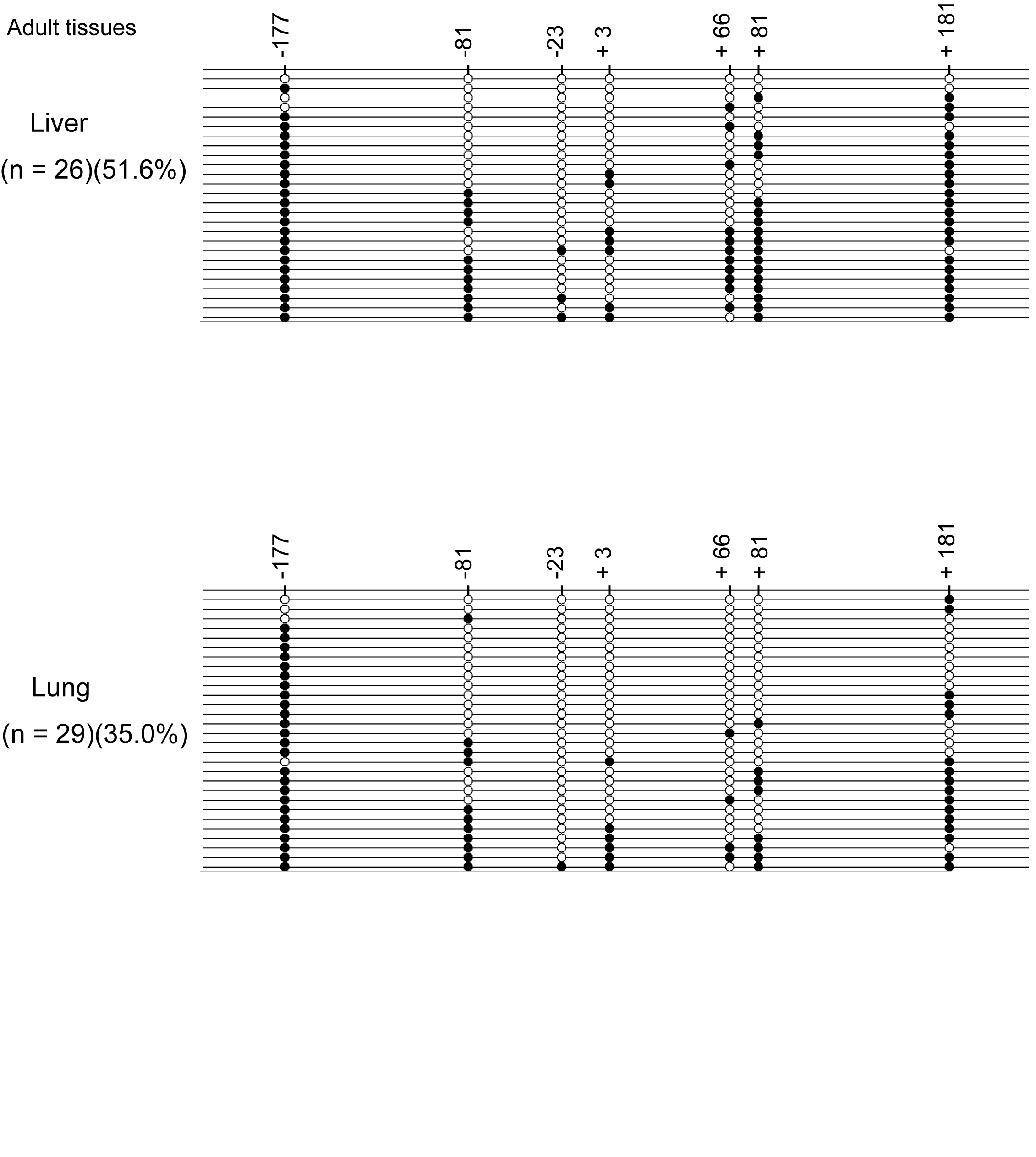

Supplement: Figure S10 — Methylation patterns of the Sytl1 promoter in somatic tissues in adult mice. Bisulfite sequencing of CpGs in the Sytl1 promoter region was carried out for liver and lung tissue cells of an adult female mouse. The analyzed region is the same as that depicted in Figure 4. The methylation level at the −23 CpG site is consistently low in both tissues. (TIF) [file pone.0019278.s010.tif]
