# Supplementary material for: Genome-Wide DNA Methylation Analysis Reveals Phytoestrogen Modification of Promoter Methylation Patterns during Embryonic Stem Cell Differentiation
Source: PLoS One. 2011 Apr 29;6(4):e19278. doi: 10.1371/journal.pone.0019278 (PMC3084807; doi:10.1371/journal.pone.0019278)
Supplement: Table S1 — Primers and Universal Probe Library probes for real-time PCR. Detailed information for Universal Probe Library probes is available online at the “Universal Probe Library Assay Design Center” of Roche Applied Science. (DOC) [file pone.0019278.s011.doc]

| **Gene** | **Universal Probe Library probe No.** | **Primer name** | **Primer sequence (5’ to 3’)** |
| --- | --- | --- | --- |
| Esr1 | #97 | Esr1-97L | gctcctaacttgctcctggac |
|  |  | Esr1-97R | cagcaacatgtcaaagatctcc |
| Esr2 | #56 | Esr2-56L | cctcagaagaccctcactgg |
|  |  | Esr2-56R | cacgcacttcccctcatc |
| Pou5f1 | #95 | Pou5f1-95L | gttggagaaggtggaaccaa |
|  |  | Pou5f1-95R | ctccttctgcagggctttc |
| T | #100 | T-100L | cagcccacctactggctcta |
|  |  | T-100R | gagcctggggtgatggta |
| Gata4 | #13 | GATA4-13L | ggaagacaccccaatctcg |
|  |  | GATA4-13R | catggccccacaattgac |
| Gata6 | #40 | GATA6-40L | ggtctctacagcaagatgaatgg |
|  |  | GATA6-40R | tggcacaggacagtccaag |
| Tbx5 | #9 | Tbx5-09L | cgaagtgggcacagagatg |
|  |  | Tbx5-09R | caccttcactttgtaactaggaaaca |
| Pdgfra | #80 | Pdgfra-80L | gtcgttgacctgcagtgga |
|  |  | Pdgfra-80R | ccagcatggtgatacctttgt |
| Fgf5 | #29 | Fgf5-29L | aaaacctggtgcaccctaga |
|  |  | Fgf5-29R | catcacattcccgaattaagc |
| Wnt3a | #76 | Wnt3a-76L | cttagtgctctgcagcctga |
|  |  | Wnt3a-76R | gagtgctcagagaggagtactgg |
| Actb | #64 | Actb-64L | ctaaggccaaccgtgaaaag |
|  |  | Actb-64R | accagaggcatacagggaca |

**Table S1. Primers and Universal Probe Library probes for real-time PCR.**
