# Supplementary material for: Genome-Wide DNA Methylation Analysis Reveals Phytoestrogen Modification of Promoter Methylation Patterns during Embryonic Stem Cell Differentiation
Source: PLoS One. 2011 Apr 29;6(4):e19278. doi: 10.1371/journal.pone.0019278 (PMC3084807; doi:10.1371/journal.pone.0019278)
Supplement: Table S2 — Primers used for bisulfite sequencing. (DOC) [file pone.0019278.s012.doc]

**Table S2**. Primers used for bisulfite sequencing.

| **Primer name** | **Sequence (5’ to 3’)** | **Ta, product size** |
| --- | --- | --- |
| Ucp1 promoter forward | GGTTGTTGAAGAGTGATAAAAGGTATTA | 54.4 ºC, 465 bp |
| Ucp1 promoter reverse | AAACTCAACCCTAAAAACAACAATAAC |
| Ucp1 enhancer forward | GGGTGTTTTGTAAATGGTGTTTTATATT | 54 ºC, 390 bp |
| Ucp1 enhancer reverse | ATTACCCAACAAAAACTTTCCTAAACT |
| Sytl1 promoter forward | GTTTGTTATAGTTTTTGGGTAAGTTTGGTA | 57 ºC, 441 bp |
| Sytl1 promoter reverse | AAACCAAAATCATAATCTTACTTACACCTC |
