# Supplementary material for: Genome-Wide DNA Methylation Analysis Reveals Phytoestrogen Modification of Promoter Methylation Patterns during Embryonic Stem Cell Differentiation
Source: PLoS One. 2011 Apr 29;6(4):e19278. doi: 10.1371/journal.pone.0019278 (PMC3084807; doi:10.1371/journal.pone.0019278)
Supplement: Table S3 — Gene ontology analysis of genes adjacent to or contained within differentially methylated regions. (DOC) [file pone.0019278.s013.doc]

**Table S3. Gene ontology analysis of genes adjacent to or contained within differentially methylated regions.**

| **Category** | **Term** | **Count** | **%** | **P-value** | **Genes** |
| --- | --- | --- | --- | --- | --- |
| GOTERM_BP_ALL | GO:0010467~gene expression | 38 | 24.5 | 1.21E-04 | ZFP46, SOX6, CBX8, TCF7L1, RPS3, ECE1, BCL11A, QTRTD1, ETV1, WDR12, MLXIP, NFATC2, KDM5A, RUNX3, DHCR24, MLLT3, ZFP36, ZFP35, EXOSC9, RCOR3, GSX2, TCFAP2B, EXOSC3, SPEN, ZFP747, NOTCH3, ZFP9, BRWD1, JMJD6, ROD1, NOTCH4, AIRE, RDBP, IRF4, TRP73, ZFP61, ZFP536, ZFP513 |
| UP_TISSUE | Embryo | 25 | 16.1 | 1.58E-02 | FAM136A, LZTFL1, TCF7L1, CCDC123, SPNB4, BCL11A, PPIL4, WDR12, ETV1, ACIN1, TMEM223, ZFP35, EXOSC9, TCFAP2B, KIF15, WDR65, KRT19, GMFG, CHPF, SULF1, RDBP, HDHD3, RIPK3, SLC40A1, FMC1 |
| UP_TISSUE | Fetal brain | 7 | 4.5 | 3.62E-02 | CHSY3, MAGI1, EXOC3, MBNL2, RCAN1, TRP73, DHCR24 |
